# Supplementary material for: Diverse Autophagosome Membrane Sources Coalesce in Recycling Endosomes
Source: Cell. 2013 Sep 12;154(6):1285–99. doi: 10.1016/j.cell.2013.08.044 (PMC3791395; doi:10.1016/j.cell.2013.08.044)
Supplement: Document S1. Article plus Supplemental Information [file mmc7.pdf]

Cell

Diverse Autophagosome Membrane Sources Coalesce in Recycling Endosomes

Claudia Puri,[1] Maurizio Renna,[1] Carla F. Bento,[1] Kevin Moreau,[1] and David C. Rubinsztein[1,*]
[1]Department of Medical Genetics, Cambridge Institute for Medical Research, University of Cambridge, Hills Road, Cambridge CB2 0XY, UK
*Correspondence: dcr1000@hermes.cam.ac.uk
http://dx.doi.org/10.1016/j.cell.2013.08.044

SUMMARY

Autophagic protein degradation is mediated by autophagosomes that fuse with lysosomes, where their contents are degraded. The membrane origins of autophagosomes may involve multiple sources. However, it is unclear if and where distinct membrane sources fuse during autophagosome biogenesis. Vesicles containing mATG9, the only transmembrane autophagy protein, are seen in many sites, and fusions with other autophagic compartments have not been visualized in mammalian cells. We observed that mATG9 traffics from the plasma membrane to recycling endosomes in carriers that appear to be routed differently from ATG16L1-containing vesicles, another source of autophagosome membrane. mATG9- and ATG16L1-containing vesicles traffic to recycling endosomes, where VAMP3-dependent heterotypic fusions occur. These fusions correlate with autophagosome formation, and both processes are enhanced by perturbing membrane egress from recycling endosomes. Starvation, a primordial autophagy activator, reduces membrane recycling from recycling endosomes and enhances mATG9-ATG16L1 vesicle fusion. Thus, this mechanism may fine-tune physiological autophagic responses.

INTRODUCTION

Macroautophagy, which we will refer to as autophagy, is a highly conserved catabolic process in which cytoplasmic proteins and organelles are engulfed by double-membrane structures called autophagosomes, which are then transported to lysosomes for degradation. Autophagy is a key regulator of many physiological and disease processes and mediates the removal of protein oligomers, mitochondria, and various pathogens. In organisms from yeast to man, autophagy is upregulated in response to nutrient deprivation to allow cells to generate energy-rich compounds from cytoplasmic macromolecules (Rubinsztein et al., 2011).

Autophagosomes are formed by the elongation and fusion of cup-shaped structures called phagophores. The biogenesis of mammalian (and yeast) autophagosomes involves two ubiquitin-like proteins, ATG12 and LC3 (ATG8) (Ohsumi and Mizushima, 2004). The earlier of the ubiquitination-like events in autophagosome biogenesis involves the conjugation of ATG12 to ATG5, after which ATG12-5 can form a complex with ATG16L1 (Mizushima et al., 2003). This ATG12-5-16L1 complex decorates prephagophore structures and phagophores but dissociates from completed autophagosomes. The elongation of the edges of the phagophore involves a second ubiquitin-like protein, LC3, an ATG8 family member, which is cleaved by ATG4 to form cytoplasmic LC3-I. Cytoplasmic LC3-I is then covalently conjugated to phosphatidylethanolamine on the phagophore membrane (where it is called LC3-II). LC3-II is specifically associated with phagophore and autophagosome membranes, and the intra-autophagosomal LC3-II is degraded in the lysosome. Thus, LC3-II levels and the numbers of LC3 vesicles correlate with autophagosome numbers (Rubinsztein et al., 2009).

The origin of the autophagosome membranes has been a major question in the field, and recent studies suggest that there may be contributions from multiple sources. Under conditions in which autophagy is induced by various forms of starvation, autophagosomes appear to be formed at the endoplasmic reticulum (ER) via structures called omegasomes (Axe et al., 2008; Hamasaki et al., 2013; Hayashi-Nishino et al., 2010; Ylä-Anttila et al., 2009) and at mitochondria (Hailey et al., 2010). Recently, we showed that plasma membrane contributes to nascent autophagosomes under both basal and autophagy induction conditions (Ravikumar et al., 2010). We found that ATG16L1 associates with clathrin-coated pits, and after internalization and uncoating, the ATG16L1-associated plasma membrane becomes associated with phagophore precursors, which mature into phagophores and then autophagosomes. Inhibition of clathrin-mediated endocytosis causes defective autophagosome formation, which is associated with impaired uptake of plasma membrane into autophagic precursors and autophagosomes (Ravikumar et al., 2010). We recently showed that the small G protein ARF6 also has a function in autophagy and that part of its effects on autophagy could be explained by the fact that it stimulates phosphatidylinositol 4,5-biphosphate (PIP_2) formation at the plasma membrane. Plasma membrane PIP_2 regulates endocytosis, and this is likely to explain part of its importance for autophagy (Moreau et al., 2012). The ATG16L1-positive phagophore precursors undergo SNARE-mediated homotypic fusion events to give rise to tubulovesicular structures, and the increase in size resulting from these fusions enhances the capacity of these structures to acquire ATG8/LC3, which marks phagophores (Moreau et al., 2011).

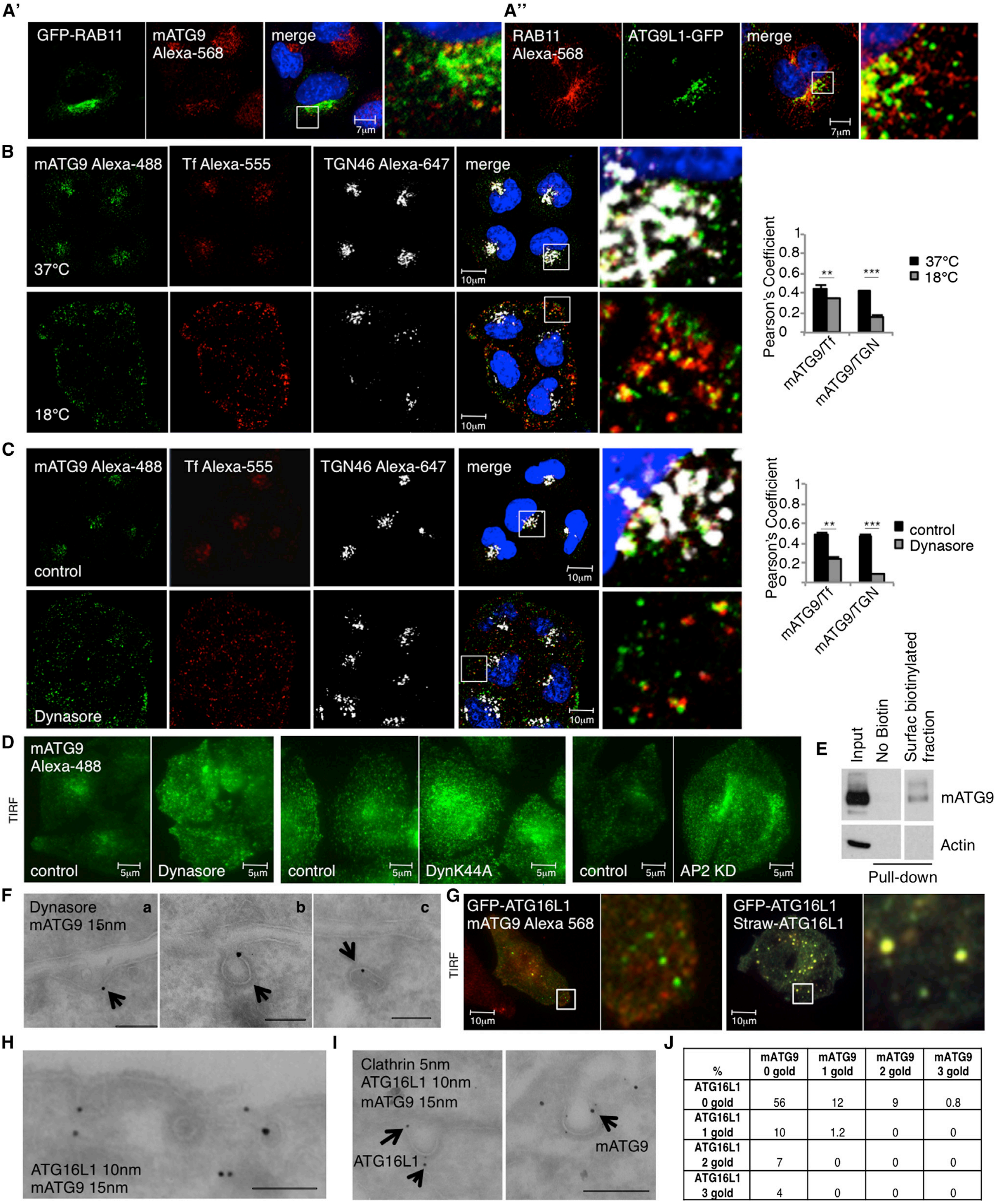

%	mATG9 0 gold	mATG9 1 gold	mATG9 2 gold	mATG9 3 gold
ATG16L1 0 gold	56	12	9	0.8
ATG16L1 1 gold	10	1.2	0	0
ATG16L1 2 gold	7	0	0	0
ATG16L1 3 gold	4	0	0	0

Figure 1. mATG9 Is Internalized by Clathrin-Mediated Endocytosis

(A′) HeLa cells transfected with pEGFP-RAB11 were fixed in basal conditions and stained for mATG9. Pearson's coefficient: 0.452 ± 0.034 (SEM).

(A″) HeLa cells were transfected with ATG9L1-pEGFP, fixed in basal condition, and labeled for RAB11. Pearson's coefficient: 0.413 ± 0.020 (SEM).

(legend continued on next page)

mATG9 is the only known multipass transmembrane protein that is necessary for optimal autophagy (Orsi et al., 2012; Young et al., 2006). Because some mATG9 is seen in autophagosomes, its itinerary may provide important clues to additional membrane sources. In yeast and in mammalian cells, mATG9 has been associated with the Golgi apparatus, suggesting that this may provide yet additional membranes for autophagosomes (van der Vaart et al., 2010; Young et al., 2006). However, these authors have argued on the basis of their recent data that this model may be overly simplistic because mammalian ATG9 (mATG9 and ATG9L1) was seen to associate with many other compartments, including recycling endosomes, early endosomes, and late endosomes (Orsi et al., 2012). Furthermore, mATG9 interactions with omegasomes and autophagosomes have been examined using live-cell imaging and fusion events were not observed, leading to the suggestion that mATG9 undergoes "dynamic" interactions with phagophores and autophagosomes without being incorporated into their membranes and that mATG9 traffics by tubulation from an existing reservoir compartment, analogous to what has been described in yeast (Mari et al., 2010; Orsi et al., 2012). It is therefore still unclear how mATG9 traffics, how it ends up associated with some LC3-positive structures if no obvious fusion events appear to occur, and whether mATG9 vesicles contribute membrane to mammalian autophagosomes. Furthermore, although distinct sources of autophagosome membranes have been proposed, it is not clear to what extent they may be mutually exclusive (possibly regulated via different autophagy signals) or whether they may coalesce and cooperate.

Here, we find that mATG9 vesicles are trafficked from the plasma membrane to the recycling endosome via clathrin-coated pits that are largely distinct from those that endocytose ATG16L1. We found that ATG16L1 was also present in the recycling compartment and participates in vesicle fusions with mATG9-postive vesicles in that site. The fusions between ATG16L1 and mATG9 are enhanced by reducing membrane egress from the recycling endosomes, a perturbation that also increases autophagosome formation. This process, which is dependent on the R-SNARE VAMP3, appears to be physiologically relevant, as starvation, an evolutionarily conserved autophagy stimulus, also reduces membrane exit from the recycling endosomes and results in increased associations of mATG9 and ATG16L1.

RESULTS

mATG9 Is Associated with Recycling Endosomes

mATG9 has been reported to be localized at the trans-Golgi network (TGN) but also in recycling endosomes (Orsi et al., 2012; Young et al., 2006). The recycling endosome is so close to the TGN that it is sometimes difficult to distinguish between the two compartments at the EM level (Puri et al., 2010). The most commonly used marker for recycling endosomes is RAB11, a small GTPase that regulates transferrin recycling through this compartment (Ren et al., 1998; Sönnichsen et al., 2000; Ullrich et al., 1996). Consistent with earlier data, we found good colocalization between mATG9 and RAB11 (Figures 1A′ and A″). Transferrin and its cognate receptor are internalized by clathrin-mediated endocytosis and recycled back to the plasma membrane through early endosomes or transferred to recycling endosomes for recycling back to the plasma membrane via a slower pathway (Maxfield and McGraw, 2004). At steady state, recycling endosomes are the predominant intracellular site at which one sees the transferrin receptor (TfR), as well as its ligand transferrin. Indeed, mATG9 colocalized well with transferrin at 37°C (Figure 1B). Likewise, mATG9 also appeared to colocalize with a TGN marker, TGN46 (Figure 1B).

In order to examine how mATG9 may arrive at recycling endosomes, we initially incubated cells at 18°C because this inhibits membrane traffic between early and recycling endosomes (Ren et al., 1998; Wilcke et al., 2000) (Figures S1A–S1C available online). As expected, the temperature block caused a peripherally biased redistribution of transferrin-positive vesicles, increased transferrin association with the early endosome

(B) HeLa cells were incubated for 1 hr at 37°C or 18°C to reach the correct temperature, loaded with transferrin Alexa-555 for 1 hr, fixed, and labeled with anti-ATG9 and anti-TGN46 (marker for Trans Golgi Network). Histogram shows Pearson's coefficient between ATG9 and transferrin (Tf) or TGN46. Error bar, SEM. ***$p < 0.001$. Inserts show merge at higher magnification.

(C) HeLa cells were incubated in serum-free medium containing Dynasore 100 μM or DMSO for 2 hr and loaded for 30 min with transferrin Alexa-555. Cells were then fixed and labeled for mATG9 and TGN46. Pearson's coefficient between mATG9 and transferrin or TGN46 was quantified. Error bar, SEM. ***$p < 0.001$. Inserts show the merge at higher magnification.

(D) Cells treated or not treated with Dynasore or transfected with Dynamin K44A mutant or AP2 siRNA were fixed and then analyzed by TIRF microscopy. Pictures are at the same magnification and brightness. Cells transfected with Dynamin mutant were identified by abnormal transferrin accumulation at the plasma membrane.

(E) HeLa cells were incubated for 1 hr at 4°C with ice-cold NHS-LC-Biotin (Pierce) solution, rinsed, and lysed in RIPA buffer. Biotinylated proteins were precipitated using streptavidin-agarose beads (Pierce) and blotted for mATG9 to assess protein localization at the plasma membrane.

(F) HeLa cells treated as in (C) were processed for immunogold labeling on cryosections and stained with anti-mATG9 antibody. Arrows show clathrin-coated structures. Scale bar in a and c, 150 nm; scale bar in b, 100 nm.

(G) HeLa cells transfected with GFP-ATG16L1 were fixed, labeled for mATG9, and then analyzed by TIRF microscopy. Control cells were cotransfected with GFP-ATG16L1 and Straw-ATG16L1. Pictures are at the same magnification and brightness. See Figure S1J for quantification.

(H) HeLa cells treated with Dynasore were processed for immunogold labeling on cryosections and double labeled with ATG16L1 (10 nm) and mATG9 (15 nm). Scale bar, 100 nm.

(I) HeLa cells treated as in (H) were processed for triple labeling on cryosections (clathrin, 5 nm; ATG16L1, 10 nm; and mATG9, 15 nm). Scale bar, 150 nm.

(J) Table shows quantification of numbers of clathrin-coated structures carrying mATG9 and ATG16L1 alone or together or neither protein. A minimum of 50 clathrin-coated structures were examined from three different experiments. We constructed a 2 × 2 table with 0 or 1–3 gold particles for mATG9 or ATG16L1 for a X^2 test to avoid statistical cells with 0 counts. $X^2 = 10.4$, df = 1, and p = 0.001.

See also Figure S1.

marker EEA1, and decreased transferrin association with the recycling endosome marker RAB11 (Figures S1A–S1C and C′). Furthermore, the 18°C incubation led to a peripherally biased dispersion of mATG9 (similar to what we observed with transferrin [Figure 1B]), increased mATG9 colocalization with the early endosome marker EEA1 (Figures S1D and D′), and reduced its colocalization with TGN46 (a marker for TGN) (Figure 1B). This suggests that the predominant effects of 18°C on mATG9 localization are not due to failed exit from the Golgi-impaired Golgi-recycling endosome trafficking, which has been described in polarized cells at 18°C (Ang et al., 2004; Gravotta et al., 2007). These results raised the possibility that most of the mATG9 that appeared to be at the TGN in basal conditions may actually be in recycling endosomes nearby and that the TGN pool of mATG9 may be quite small. Furthermore, these data suggest that most of the mATG9 in the recycling endosomes comes from the early endosomes/plasma membrane rather than from the TGN. (The decreased transferrin-mATG9 colocalization at 18°C is very likely due to the known reduction of transferrin uptake that occurs at reduced temperature [Figure 1B].)

mATG9 Is Internalized by Clathrin-Mediated Endocytosis

The experiments described above reveal that a pool of mATG9 is trafficking with transferrin. Because transferrin enters the cell by clathrin-mediated endocytosis, we examined whether mATG9 is trafficked to the plasma membrane and then internalized by clathrin-mediated endocytosis. First, we acutely inhibited endocytosis by treating cells with the dynamin inhibitor Dynasore (Thompson and McNiven, 2006). Dynasore treatment caused a redistribution of mATG9 from a perinuclear concentration to a more dispersed localization, and this was associated with decreased colocalization of mATG9 with transferrin or a Golgi marker (Figure 1C). Total internal reflection fluorescence (TIRF) microscopy, which enables high-resolution assessment of molecules close to the plasma membrane, suggested that mATG9 redistributed close to the plasma membrane in Dynasore-treated cells (Figure 1D). A similar redistribution of mATG9 from its normal perinuclear localization close to the plasma membrane was seen in cells overexpressing a dominant-negative dynamin mutant or small interfering RNA (siRNA) to the clathrin adaptor AP2 (to block clathrin-dependent endocytosis) (Figures 1D and S1E–S1G). The presence of mATG9 on the plasma membrane was confirmed by cell surface biotinylation, followed by immunoprecipitation (Figure 1E). Moreover, electron microscopy (EM) analysis showed mATG9 on clathrin-coated structures on the plasma membrane (Figures 1Fa–1Fc). As a control, we showed that almost no clathrin labeling was observed in cells transfected and labeled with GFP (Figure S1H). In addition to clathrin-coated structures, EM also revealed mATG9 associated with plasma membrane, endocytic structures, autophagic structures, and Golgi (Figure S1I).

ATG16L1 and mATG9 Are Not Internalized by the Same Clathrin-Coated Structures

Because both ATG16L1 (Ravikumar et al., 2010) and mATG9 are associated with clathrin-coated pits, we wondered whether they were in the same structures and followed the same internalization routes. To address this question, we transfected HeLa cells with GFP-ATG16L1, fixed, labeled for endogenous mATG9, and analyzed by TIRF microscopy, which assesses structures within 200 nm of the plasma membrane (Figures 1G and S1J). Note that clathrin-coated structures on the plasma membrane are 100–150 nm in diameter (Stoorvogel et al., 1996). In the same experiment, control cells were cotransfected with mStrawberry-ATG16L1 and GFP-ATG16L1. This experiment revealed that the mATG9 and ATG16L1 signals were almost completely distinct. The control experiment showed almost complete overlap of the mStrawberry-ATG16L1 and GFP-ATG16L1 (Figures 1G and S1J). In order to analyze the clathrin-coated structures carrying these two proteins with greater resolution, we treated cells with Dynasore to recruit as much as possible ATG16L1 and mATG9 to the plasma membrane and then performed immunogold labeling on cryosections to observe the two proteins. Remarkably, at the EM level, mATG9 and ATG16L1 appeared to associate with two different pools of clathrin-coated structures (Figures 1H–1J). mATG9 and ATG16L1 appear to be internalized via different routes because mATG9 was associated with EEA1-positive early endosomes, whereas ATG16L1 was not seen with EEA1 (consistent with what we have reported previously [Moreau et al., 2011; Ravikumar et al., 2010]) (Figure S1K). We confirmed this observation using the RAB5 constitutive-active mutant Q79L, which induces aberrant fusion of early endosomes (Stenmark et al., 1994) and has been used widely to enable better resolution of proteins associated with early endosomes. This RAB5 mutant colocalizes only with mATG9 and not with ATG16L1 (Figure S1L).

ATG16L1 Vesicles Are Seen in the RAB11 Compartment

Because mATG9 vesicles appear to arrive at recycling endosomes via endocytosis, we investigated whether ATG16L1 vesicles also localized to that compartment. We performed EM studies to identify possible intracellular sites associated with ATG16L1 and observed ATG16L1-positive tubulovesicular structures (probably phagophore precursors) localizing close to the Golgi (Figure 2A). These ATG16L1-positive structures are associated with endocytosed cholera toxin (Figure 2A), which binds to the plasma membrane and traffics to recycling endosomes and the Golgi (Bonifacino and Rojas, 2006; Ravikumar et al., 2010). Because the ATG16L1 tubulovesicular structures appear to be distinct from the Golgi, we tested whether ATG16L1 trafficked through recycling endosomes. ATG16L1 colocalized with RAB11 (Figures 2B–2D). EM analysis showed that ATG16L1 and RAB11 localized in the same tubulovesicular structures (Figure 2C). We have used very low levels of overexpressed ATG16L1 to avoid artifacts (Ravikumar et al., 2010) but to enable efficient visualization of those vesicles.

We have previously shown that low levels of exogenous ATG16L1 decorate early autophagic structures and colocalize with ATG5 and ATG12 (Moreau et al., 2011; Ravikumar et al., 2010). Indeed, ATG12 also colocalized with RAB11 (Figure S2A).

Homotypic fusion of ATG16L1 vesicles is an important step that increases the size of these structures and facilitates their progression to the phagophore stage (Moreau et al., 2011). Accordingly, we tested whether homotypic fusions of ATG16L1 vesicles associated with the RAB11 compartment. Interestingly,

live-imaging analysis of ATG16L1 and RAB11 confirmed that such homotypic fusions occurred, and there also appeared to be progressive recruitment of RAB11 (vesicles from recycling endosomes) to the ATG16L1 (preautophagosome) vesicles (Figure 2D and Movie S1), suggesting that the recycling compartment may provide membranes to preautophagosomal structures.

ATG16L1 Colocalizes with mATG9 in Recycling Endosome Compartment

Because ATG16L1 and mATG9 are both localized to recycling endosomes, we tested and confirmed that they colocalize together with RAB11 (Figure S2B). This was consistent with EM data showing colocalization of ATG16L1 and mATG9 on structures that are associated with the transferrin receptor (Figure 2E). Importantly, this colocalization is occurring prior to the phagophore stage because such colocalizations appear in some vesicles that are LC3 negative (Figure 2F). Although previous studies have failed to see proper fusions between mATG9-containing vesicles and autophagosome-related structures, these did not study ATG16L1 phagophore precursors (Orsi et al., 2012). However, we observed authentic and long-lasting fusions between mATG9 and ATG16L1-positive structures, suggesting that mATG9 vesicles and associated membranes mix with ATG16L1-positive phagophore precursors and that mATG9 becomes associated with autophagosome precursors at this early prephagophore stage (Figure 2G and Movie S2). The colocalization of mATG9 and ATG16L1 in the recycling endosomes raised the possibility that these fusion events may be occurring in that compartment.

Temperature Block Inhibits mATG9-ATG16L1 Vesicle Fusion and Autophagosome Formation

Our data suggest that the recycling endosome is the site of fusion between mATG9 and ATG16L1 vesicles and led us to hypothesize that membrane flow in and out of the recycling compartment may influence mATG9-ATG16L1 fusions and autophagy. Accordingly, we initially studied the effect of reducing the temperature to 18°C because this inhibits membrane traffic between early and recycling endosomes (Ren et al., 1998; Wilcke et al., 2000). The most common method used to study autophagy is to assay LC3-II levels, which correlates with autophagosome numbers. LC3-II changes can result from altered autophagosome formation and/or degradation. These possibilities can be deconvoluted by studying cells in the absence and presence of lysosomal inhibitors like Bafilomycin A_1 or NH_4Cl, which inhibit autophagosome degradation and hence allow one to assess autophagosome formation rates (Rubinsztein et al., 2009). The temperature block (18°C) that inhibits membrane traffic between early and recycling endosomes (Ren et al., 1998; Wilcke et al., 2000) (Figures S1A–S1C) and which causes endocytosed transferrin to accumulate mostly in early endosomes (Figures S1B and S1C) also clearly impairs autophagosome formation as measured on LC3-II blots (Figures 3A–3C). Cells incubated at 18°C also had more numerous but smaller ATG16L1 vesicles (Figures 3D and 3E)—which is compatible with decreased efficiency and/or frequency of ATG16L1 vesicle homotypic fusion events (Moreau et al., 2011)—and caused accumulation of ATG16L1 in recycling endosomes but did not lead to a change in its negligible abundance in early endosomes (note that the low level of colocalization we see may be "background" due to labeling of adjacent structures) (Figures 3F and 3G). In addition, the temperature block decreased the extent to which ATG16L1 and mATG9 colocalized and decreased the sizes of both mATG9 and ATG16L1 vesicles (Figures 3H and S3A for separate channels), suggesting that this treatment impaired homotypic and heterotypic fusion of ATG16L1 and mATG9 vesicles.

Because decreased temperature has diverse effects, one cannot draw very strong conclusions that the 18°C effects are specifically due to decreased membrane flow into the recycling compartment. Accordingly, we aimed to test the converse situation by reducing membrane egress from the recycling compartment by overexpressing RAB11 (Ren et al., 1998) or by inhibiting Myosin Vb by overexpressing its tail domain (Lapierre et al., 2001). Both RAB11 and Myosin Vb tail overexpression enhanced autophagosome formation (LC3-II levels in the presence or absence of Bafilomcyin A_1) (Figure 3I). Autophagy regulates the proportion of cells with mutant huntingtin Q74 (HTT) aggregates by regulating mutant HTT clearance, and the percentage of cells with aggregates correlates inversely with autophagic activity (Luo and Rubinsztein, 2010; Ravikumar et al., 2004). The proportion of cells with mutant HTT aggregates was reduced in cells transfected with RAB11 or the Myosin Vb tail (Figure S3B). Overexpression of RAB11 and Myosin Vb tail also increased the size of ATG16L1 vesicles (Figures 3J and S3C) (consistent with increased homotypic fusion of ATG16L1 vesicles), increased the colocalization of ATG16L1 with the transferrin (Figures 3K and S3D) (consistent with more ATG16L1 in the recycling compartment), and increased the colocalization of ATG16L1 and mATG9 (Figures 3L and S3E) (consistent with enhanced heterotypic fusion of mATG9 and ATG16L vesicles). Furthermore, homotypic ATG16L1 interactions were also enhanced in real time when the cells overexpressed RAB11 or the Myosin Vb tail (Figures S3F and S3G).

Starvation Decreases Recycling and Increases ATG16L1 and mATG9 Colocalization

We examined the links between autophagy induction, recycling endosomes, homotypic ATG16L1 fusion, and heterotypic ATG16L1-mATG9 fusion during starvation, an evolutionarily conserved autophagic stimulus. Amino acid and serum starvation decreased transferrin recycling, similar to what is observed with RAB11 or Myosin Vb tail overexpression (Lapierre et al., 2001; Ren et al., 1998; Wilcke et al., 2000) (Figure S3H). Again, similar to RAB11 or Myosin Vb tail overexpression, starvation increased ATG16L1 vesicle interactions in real time, consistent with what we reported previously (Moreau et al., 2011) (Figure S3I and Movies S3 and S4). Likewise, starvation increased the colocalization of ATG16L1 with transferrin (Figure 3M) and increased the colocalization of ATG16L1 with mATG9 (Figure 3N).

VAMP3 Mediates ATG16L1-mATG9 Fusion

These data suggest that mATG9 vesicles likely travel from early endosomes to recycling endosomes where they meet ATG16L1-containing vesicles, which arrive at recycling endosomes via a

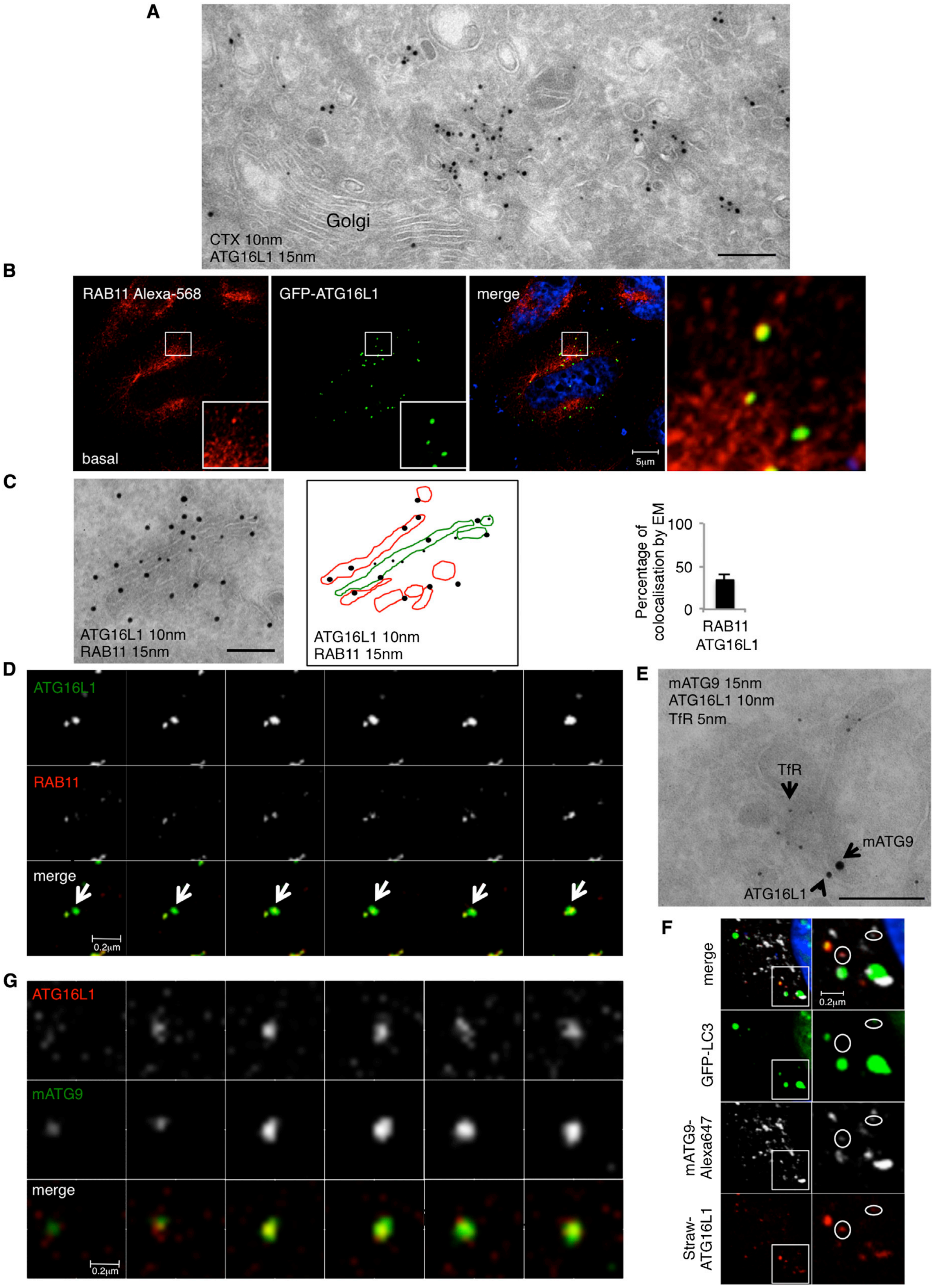

(legend on next page)

route that does not appear to involve a significant residence time in early endosomes. The fusion of these vesicles appears to be rate limiting for autophagosome formation. In order to clarify the itineraries of these vesicles and their trafficking routes more robustly, we needed to identify further molecular regulators of their fusion. Because intracellular vesicular fusions are generally SNARE dependent, we tested and confirmed that this was the case for ATG16L1-mATG9 fusions because *N*-ethylmaleimide (NEM), which abolishes SNARE fusion (Jahn and Scheller, 2006; Moreau et al., 2011; Galli et al., 1994), reduced the colocalization in cells between ATG16L1 and mATG9 (Figure 4A) and impaired the fusion of these two vesicle populations in vitro using postnuclear supernatants isolated from either Strawberry-ATG16L1- or ATG9L1-GFP-expressing cells (Figures 4B–4D). Typically, SNARE complexes comprise three molecules with highly conserved glutamine (Q) residues in the SNARE motif and one with a highly conserved arginine (R) residue in this domain, namely Qa-, Qb-, Qc-, and R-SNAREs (Jahn and Scheller, 2006). In order to identify such SNAREs pertinent to ATG16L1 and mATG9 vesicle fusion, we started screening for the levels of the autophagy substrate p62 (Renna et al., 2011) in cells where different R-SNAREs were knocked down with siRNAs. VAMP3 knockdown resulted in the strongest elevation of p62 levels (Figure 5A). We confirmed that VAMP3 indeed regulated autophagy by showing that it decreased autophagosome numbers (LC3-II levels on western blots versus actin and a 60% reduction in the number of LC3-positive vesicles [autophagosomes]), which could be accounted for by impaired autophagosome biogenesis (LC3-II levels in the presence of Bafilomycin A_1) (Figures 5B, 5C, S4A, and S4B).

VAMP3 localizes in early endosomes (EEA1 positive) and recycling endosomes (RAB11 positive) (Figure 5D), as described previously (Yamazaki et al., 2013; Prekeris et al., 1998), suggesting that it was in appropriate compartments for regulating mATG9-ATG16L1 vesicle fusions. A temperature block at 18°C, which impairs trafficking between early and recycling endosomes, did not affect the colocalization between mATG9 and VAMP3, suggesting that the two proteins are cotrafficking (Figure 5E), but reduced the colocalization between ATG16L1 and VAMP3 (Figures 5E and S4C) in a manner reminiscent to what we observed with mATG9 and ATG16L1 (Figure 3H). These data strengthen the likelihood of a connection between VAMP3 and mATG9-ATG16L1 and support the hypothesis that VAMP3 is trafficking with mATG9 from early endosomes to recycling endosomes where ATG16L1 localizes.

VAMP3 knockdown did not affect the morphology of the recycling endosomes (marked by RAB11) (Ren et al., 1998) or the endocytosis of transferrin (Figures S4D and S4E), suggesting that it did not grossly affect endocytic trafficking.

However, VAMP3 knockdown caused the accumulation of mATG9 in early endosomes (EE) and reduced its localization in recycling endosomes (RE) (Figures 6A–6C). In contrast to what we observed with mATG9, knockdown of VAMP3 caused the accumulation of ATG16L1 in recycling endosomes but did not lead to a change in its negligible abundance in early endosomes (note that the low level of colocalization we see may be ‘‘background’’ due to labeling of adjacent structures) (Figures 6D–6F). VAMP3 knockdown reduced the colocalization of mATG9 and ATG16L1 (Figure 7A), and live-cell imaging demonstrated that coalescence between ATG16L1 and mATG9 was abrogated by VAMP3 knockdown as compared to control cells (Figures S5A and S5B; see also Movies S5 and S6) without affecting homotypic fusion of ATG16L1 vesicles (Figure 7B). Consistent with these data, VAMP3 knockdown impaired in vitro fusion of ATG16L1 and mATG9 vesicles (vesicles probably derived from recycling and early endosomes, respectively) (Figure 7C).

DISCUSSION

Previous studies have shown that loss of mATG9 compromises mammalian autophagy, suggesting that this protein is a critical part of the autophagy apparatus (Orsi et al., 2012). Its role in autophagy has, however, been elusive. As the only known multipass transmembrane ATG protein, it has been assumed that it may play a role in lipid delivery to nascent autophagosomes. However, despite having some colocalization with LC3-positive structures, mATG9 was previously found to have less colocalization with autophagic markers than with other organelles (Longatti et al., 2012; Orsi et al., 2012). This observation could be interpreted as suggesting that mATG9 has a complex itinerary and that it fuses with autophagosomes at some stage of their biogenesis. However, based on studies examining LC3- or DFCP1-positive structures, mATG9 did not seem to fuse with these structures but instead circled around them, leaving the question open whether mATG9 contributed membrane to autophagosomes or whether it had some other activities.

Figure 2. ATG16L1 Is Found in Recycling Endosomes

(A) HeLa cells transfected with GFP-ATG16L1 for 24 hr were incubated for 15 min with 2.5 µg/ml with HRP-cholera toxin subunit B at 4°C and then for 10 min at 37°C. Cells were then fixed and treated for immunogold labeling on cryosections. ATG16L1 was detected with anti-ATG16L1 antibody (15 nm), and cholera toxin was detected using anti-HRP antibody. Scale bar, 150 nm.

(B) HeLa cells were transiently transfected with GFP-ATG16L1 for 20 hr. Cells in basal conditions were fixed and labeled with anti-RAB11 antibody.

(C) HeLa cells transfected with mStrawberry-ATG16L1 (20 hr) and starved (4 hr) were processed for immunogold labeling on cryosections. Double labeling was performed with rabbit anti-ATG16L1 (10 nm gold) and rabbit anti-RAB11 (15 nm gold). Scale bar, 150 nm. Schematic diagram shows membrane outlines from EM. Green lines indicate structures positive for both ATG16L1 and RAB11, whereas red lines are only positive for RAB11. Histogram shows the percentage by EM of ATG16L1 tubulovesicular structures that carry RAB11. 281 tubulovesiclular structures from different experiments were analyzed. Error bars, SD.

(D) HeLa cells were transfected with EGFP-ATG16L1 and mCherry-RAB11, and 5 min movies were recorded. Representative images from movies are shown.

(E) HeLa cells were loaded with mouse anti-transferrin receptor antibody (5E9C11) for 30 min and fixed for immunogold labeling for cryosections. The sample was labeled with rabbit anti-mouse antibody to recognize transferrin receptor (5 nm), anti-ATGL116 (10 nm), and anti-mATG9 (15 nm). Scale bar, 150 nm.

(F) HeLa cells were transfected with pmStrawberry-ATG16L1 and ATG9L1-pEGFP, and 5 min movies were recorded. Representative images from the movies are shown.

(G) HeLa cells were transfected with pmStrawberry-ATG16L1 and pEGFP-LC3, starved for 2 hr, fixed, and labeled for mATG9.

See also Figure S2 and Movies S1 and S2.

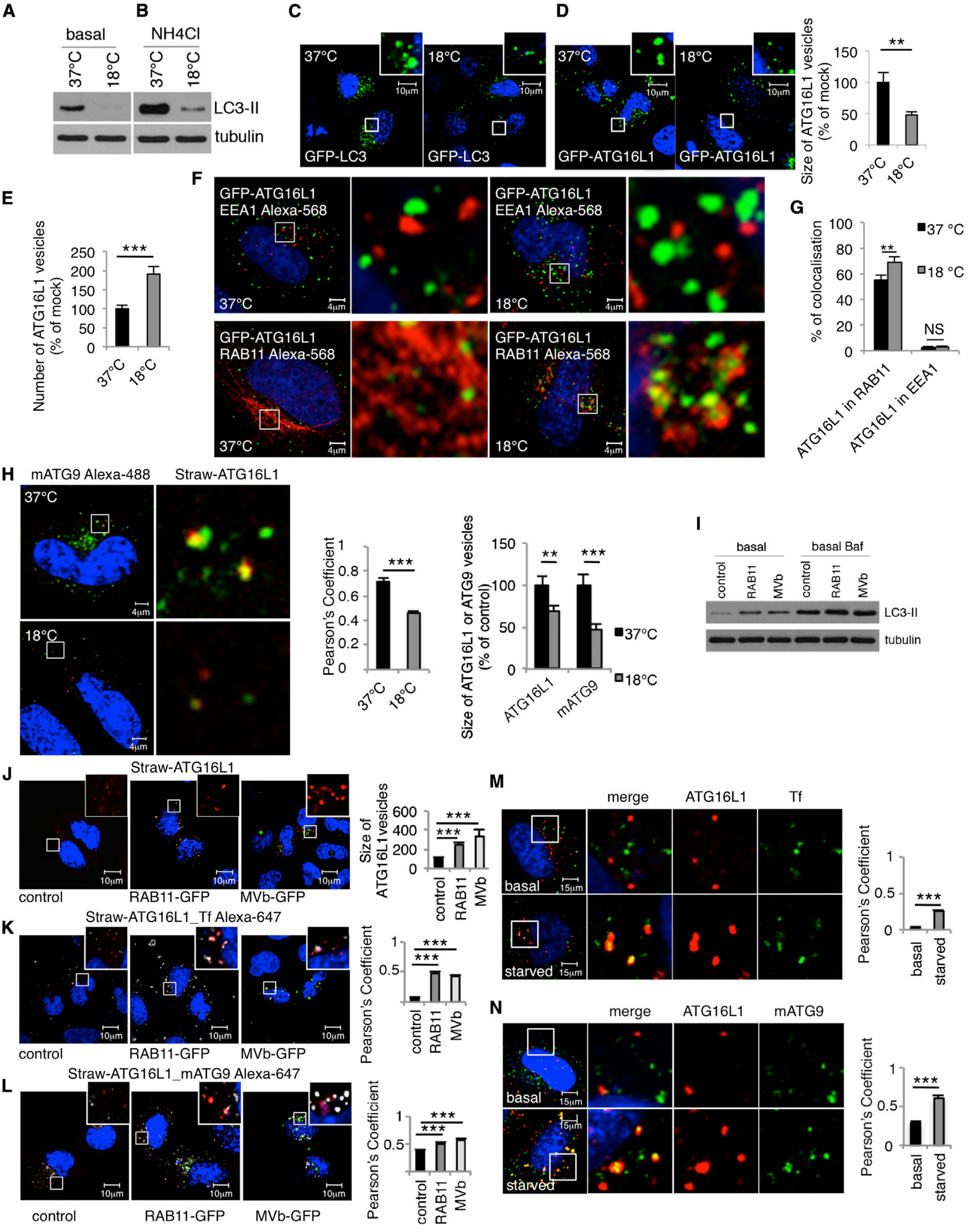

(legend on next page)

Our data suggest that mATG9 does indeed follow a complex itinerary and also becomes associated with nascent autophagosomes. Because mATG9 is a transmembrane protein, it is likely that it is incorporated into autophagosomes precursors with some membrane from its originating compartment. Previously, we showed that the plasma membrane is important for autophagosome biogenesis by contributing membrane to ATG16L1-positive phagophore precursors (Moreau et al., 2011; Ravikumar et al., 2010). Interestingly, we found very low levels of colocalization between ATG16L1 and EEA1, suggesting that the ATG16L1 vesicles were routed away from early endosomes soon after clathrin uncoating (Moreau et al., 2011; Ravikumar et al., 2010). Here, we have found that mATG9 also traffics via the plasma membrane. In this case, mATG9 appears to be localized to a largely distinct pool of clathrin pits compared to ATG16L1, and although both proteins end up in recycling endosomes, mATG9 is trafficked to recycling endosomes via EEA1-positive early endosomes, whereas ATG16L1 has minimal residence in early endosomes (supported by both temperature block and VAMP3 knockdown experiments). The observation that mATG9 and ATG16L1 are trafficking via distinct routes is entirely consistent with the idea that they are internalized via largely distinct clathrin coats. There is a precedent for this type of process because it has been previously shown that different plasma membrane receptors are frequently endocytosed via different clathrin-coated structures (Tosoni et al., 2005; Warren et al., 1997, 1998; Wiley, 1988).

It is likely that some plasma membrane mATG9 ends up being trafficked from early to recycling endosomes for fusion with ATG16L1. Although we cannot exclude other sources for early endosomal mATG9, our temperature block experiments suggest that mATG9 in early endosomes is not predominantly derived from the Golgi, as this protein accumulates in early endosomes rather than the Golgi at 18°C.

Our data suggest that the recycling endosome is where true fusions occur between distinct ATG16L1 and mATG9-positive structures. The fusion event occurs at the prephagophore stage, as it involves ATG16L1-positive, LC3-negative vesicles. The coalescence of mATG9 and ATG16L1 depends on SNARE function, and we have identified VAMP3 as one of the critical mediators. The mATG9-ATG16L1 fusions are distinct from the homotypic ATG16L1 fusions, as the former, but not the latter, requires VAMP3. VAMP3 appears to colocalize first with mATG9 in early endosomes (Figure 5E) and then likely fuses with partner SNAREs on ATG16L1 in recycling endosome-associated structures, resulting in colocalization with both proteins. This model is consistent with our observations with the 18°C temperature block, which inhibits membrane trafficking between early and recycling endosomes (Ren et al., 1998; Wilcke et al., 2000), as this treatment decreased the association of VAMP3 with ATG16L1 (Figures 5E and S4C), but not with mATG9 (Figure 5E), and impaired the colocalization between ATG16L1 and mATG9 (Figure 3H).

Although VAMP3 has previously been linked to autophagy, it is important to clarify that Fader et al. (2009) suggested that VAMP3 is involved in a much later stage of the pathway—the fusion of already-completed autophagosomes with multivesicular bodies to form amphisomes. This is not the stage of autophagosome biogenesis we are concerned with—we are describing the processes and structures preceding the formation of LC3-positive autophagosomes. It is also worth noting that Fader et al. (2009) do not formally demonstrate that VAMP3 is the relevant SNARE, as they use tetanus toxin for their experiments, and this toxin inactivates many SNAREs.

Our data reveal that membrane traffic from early to recycling endosomes is crucial for autophagosome formation and that the recycling compartment is not merely responsible for recycling of plasma membrane receptors but also serves as a station

Figure 3. mATG9 and ATG16L1 Meet in Recycling Endosomes

(A and B) (A) HeLa cells were incubated for 4 hr at 37°C or 18°C and processed for LC3II western blot. Some cells were incubated overnight with 20 mM NH_4Cl in full medium (to block lysosomal degradation) at 37°C or 18°C (B).

(C) HeLa cells were transfected with pEGFP-LC3 for 20 hr and incubated for 4 hr at 37°C or 18°C.

(D and E) (D) HeLa cells were transfected with pEGFP-ATG16L1 for 20 hr and incubated for 4 hr at 37°C or 18°C. The size and the number (E) of vesicles were scored (a minimum of 20 cells were examined for each condition). Error bar, SEM. ***$p < 0.001$ and **$p < 0.01$.

(F and G) (F) HeLa cells were transfected with pEGFP-ATG16L1 for 20 hr, incubated for 4 hr at 37°C or 18°C, and labeled with EEA1 or RAB11 to visualize early endosomes or recycling endosomes. Histogram in (G) shows the amount of colocalization of ATG16L1 in EEA1 or in RAB11 compartment (Manders' coefficient). Error bar, SEM. NS, not significant; **$p < 0.01$.

(H) HeLa cells were transfected with pmStrawberry-ATG16L1 for 20 hr, fixed, and labeled for mATG9. The histograms show quantification of colocalization and the size of the vesicles labeled with ATG16L1 and mATG9 at 37°C or 18°C. Error bar, SEM. ***$p < 0.001$ and **$p < 0.01$.

(I) HeLa cells were transfected with pEGFP-RAB11 or pEGFP-Myosin Vb tail (MVb) and incubated during the last 16 hr with Bafilomycin A_1 (Baf) or DMSO and processed for LC3-II western blot. Control cells were transfected with pEGFP empty vector.

(J) HeLa cells were transfected with ATG16L1-mStrawberry and RAB11-EGFP or MVb tail-EGFP. The size of ATG16L1 vesicles was scored in RAB11 and MVb tail overexpression conditions. The inserts show just the ATG16L1 vesicles as quantified in histogram. Error bar, SEM. ***$p<0.001$.

(K) HeLa cells were transfected as in (C) and loaded with transferrin Alexa-647 for 30 min. Histogram and the inserts show the correlation between ATG16L1 and the loaded transferrin (Pearson's coefficient). Error bar, SEM. ***$p < 0.001$.

(L) HeLa cells were transfected as in (C) and labeled for mATG9. Histogram and the inserts show the correlation between ATG16L1 and mATG9 in RAB11 and MVb tail overexpression conditions (Pearson's coefficient). Error bar, SEM. ***$p < 0.001$.

(M) HeLa cells were transfected with mStrawberry-ATG16L1 for 20 hr, starved in HBSS for 4 hr, or maintained in full medium, loaded with transferrin Alexa-488 (Tf). Pictures and histogram show the correlation between ATG16L1 and transferrin in basal and starving condition (Pearson's coefficient). Error bar, SEM. ***$p < 0.001$.

(N) HeLa cells were transfected with mStrawberry-ATG16L1 for 20 hr, starved in HBSS for 4 hr, or maintained in full medium and then fixed and labeled with anti-mATG9 antibody. Pictures and histogram show the correlation between ATG16L1 and mATG9 in basal and starved cells (Pearson's coefficient). Error bar, SEM. ***$p < 0.001$.

See also Figure S3 and Movies S3 and S4.

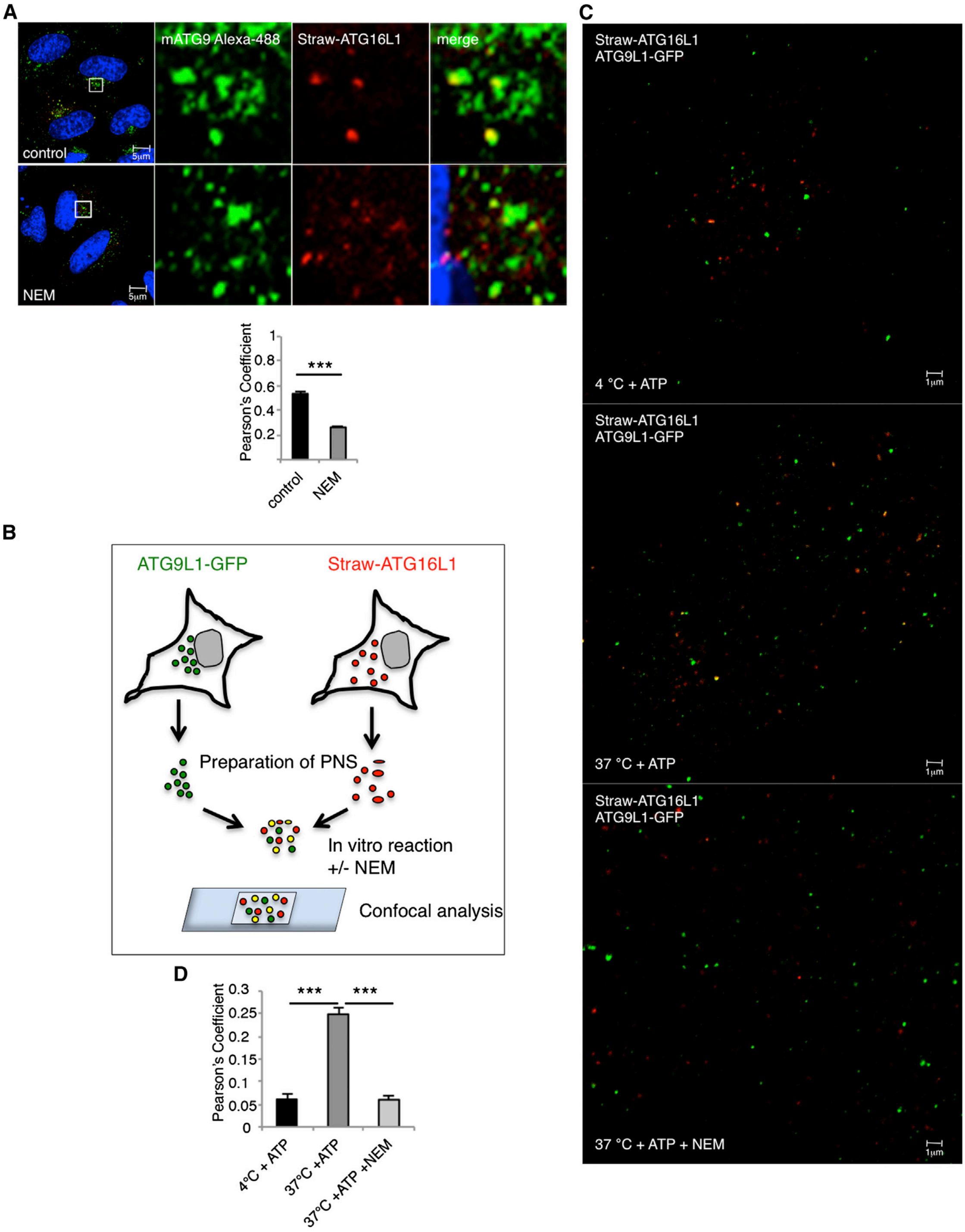

Figure 4. mATG9/ATG16L1 Vesicle Fusion Is SNARE Dependent

(A) HeLa cells were transfected with mStrawberry-ATG16 for 20 hr and treated for 10 min with N-ethylmaleimide (NEM; 100 μM) in full medium, fixed, and stained for mATG9. Histogram shows correlation between mATG9 and ATG16L1 (Pearson's coefficient). Error bar, SEM. ***$p < 0.001$.

(B) Schematic diagram of the in vitro fusion assay of ATG16L1 and mATG9 vesicles. Postnuclear supernatants (PNS) of cells expressing mStrawberry-ATG16L1 were mixed with PNS from cells expressing ATG9L1-GFP for 1 hr in the presence of ATP, ATP and NEM at 37°C, or with ATP on ice. The samples were fixed and mounted on a microscope slide with Mowiol 4-88 for confocal analysis.

(C and D) (C) Representative fields of in vitro fusion assay of ATG16L1 and mATG9 vesicles. The quantification in (D) was performed on ten fields (similar to that shown in the figure) per experiment. The quantification is expressed as Pearson's coefficient. Error bar, SEM. **$p < 0.001$.

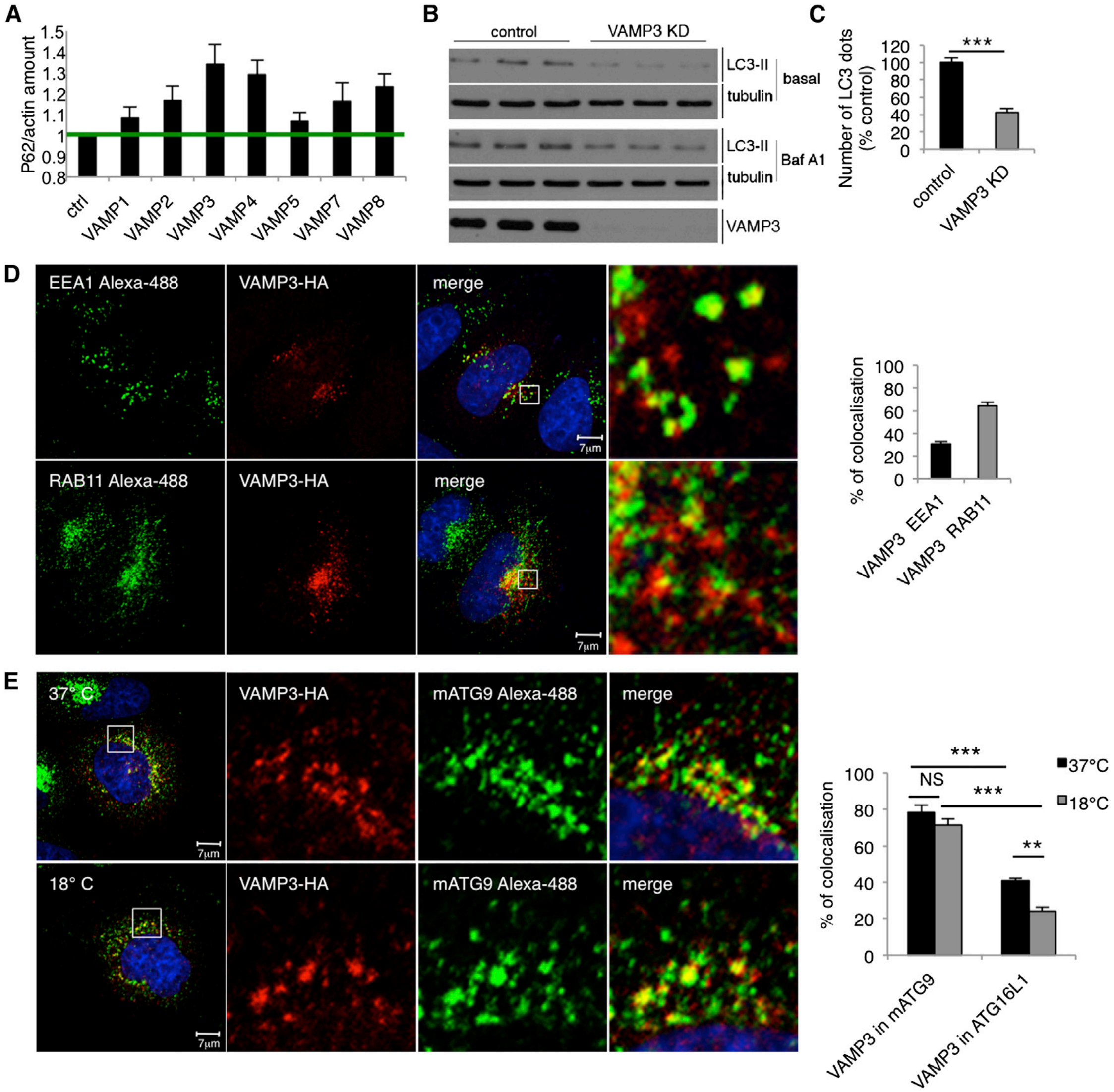

Figure 5. VAMP3 Regulates Autophagy

(A) HeLa cells were transfected with siRNAs for different R-SNAREs, and we assessed the levels of the autophagy substrate p62 versus actin.

(B) HeLa cells transfected with control or VAMP3 siRNA (oligo A) for 4 days were treated during the last 16 hr with Bafilomycin A1 (Baf A1) or DMSO (basal) and processed for LC3-II western blots.

(C) Endogenous LC3 immunocytochemistry in HeLa cells transfected with control or VAMP3 siRNA for 4 days. The histogram shows the number of vesicles (LC3 positive) in a minimum of 30 cells. Error bar, SEM. ***$p < 0.001$.

(D) HeLa cells were transfected with VAMP3-HA for 20 hr and stained for EEA1 or RAB11. Histogram shows colocalization between VAMP3-HA and EEA1 or VAMP3-HA and RAB11 (Manders' coefficient). Error bar, SEM.

(E) HeLa cells were transfected for 20 hr with VAMP3-HA, incubated for 1 hr at 37°C or 18°C, fixed, and labeled for HA and mATG9. Histogram shows the percentage of colocalization between VAMP3 with mATG9 (Manders' coefficient). Error bar, SEM. NS, not significant.

See also Figure S4.

in the early stages of autophagosome biogenesis where mATG9 and ATG16L1 meet. The observation that VAMP3 knockdown has no obvious effect on transferrin recycling (compared to the dramatic consequences of the 18°C temperature block) suggests either that the fusion of the mATG9 and ATG16L1 compartments may have a higher dependency on this R-SNARE than conventional endocytic trafficking or that VAMP3 may be mediating selective fusion of components of the early endosomal and recycling endosome compartments rather than "bulk" fusion. The relevant partner, Q-SNAREs, regulating this event will need to be elucidated in future studies (and may be difficult to pinpoint because some SNAREs participate in multiple distinct fusion events as a function of their partners [Jahn and Scheller, 2006]). However, the identification of VAMP3 allowed us to propose that the coalescence between ATG16L1 and mATG9 in recycling endosomes mediated by VAMP3 is likely a critical event

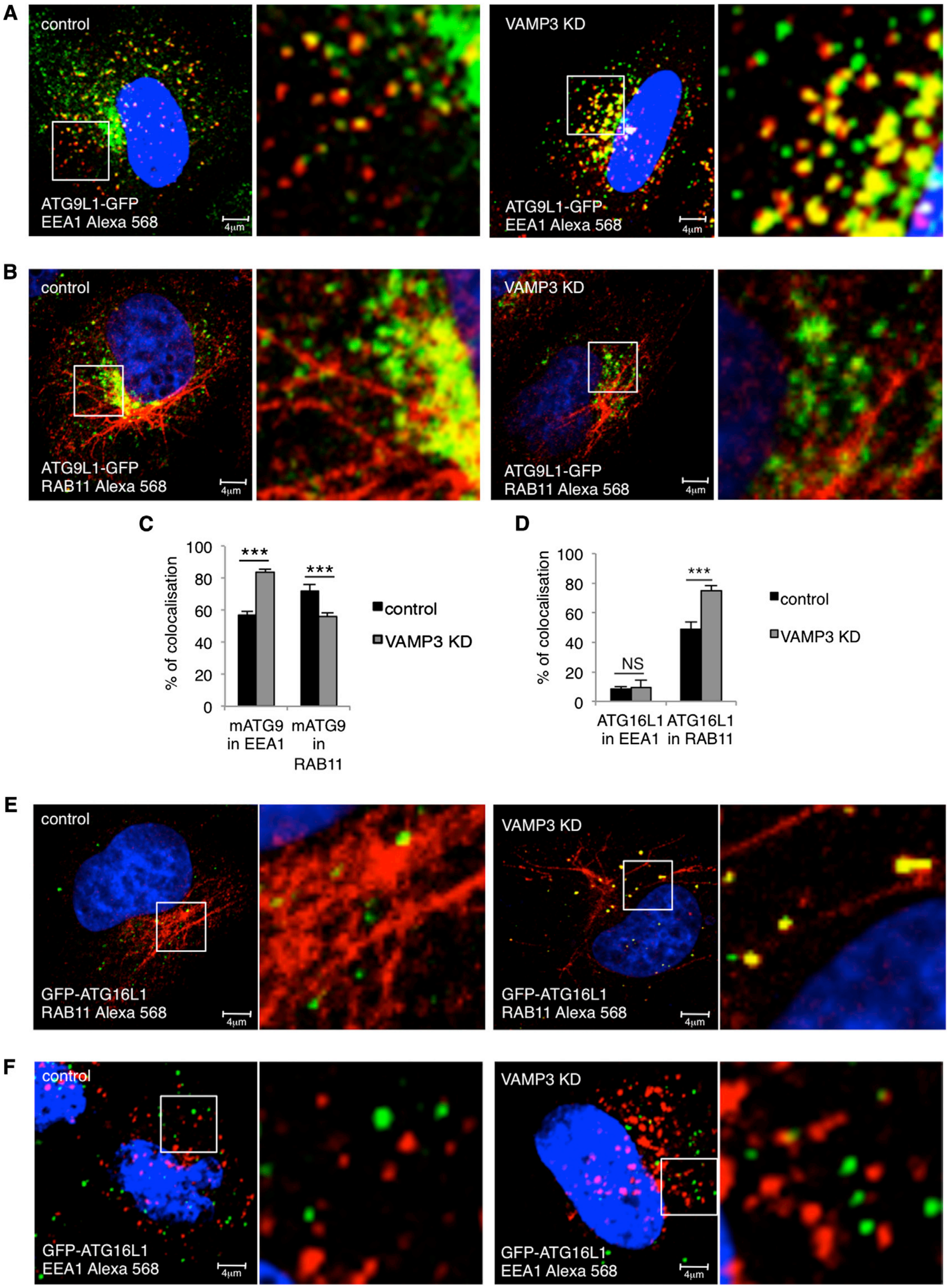

Figure 6. VAMP3 Regulates mATG9 and ATG16L1 Trafficking

(A–C) HeLa cells transfected with control, VAMP3 siRNA for 4 days were transfected during the last 20 hr with ATG9L1-pEGFP and labeled with anti-EEA1 (for early endosomes) or anti-RAB11 (for recycling endosomes). The histogram in (C) shows the quantification of the colocalization of mATG9 in EEA1 or RAB11 (Manders' coefficient). Error bars, SEM. ***p < 0.001.

(D–F) HeLa cells were treated as in (A)–(C) and transfected during the last 20 hr with pEGFP-ATG16L1 and labeled with anti-EEA1 (early endosomes) or anti-RAB11 (recycling endosomes). Histogram in (D) shows quantification of the colocalization of ATG16L1 in EEA1 or RAB11 (Manders' coefficient). Error bars, SEM. ***p < 0.001; NS, not significant.

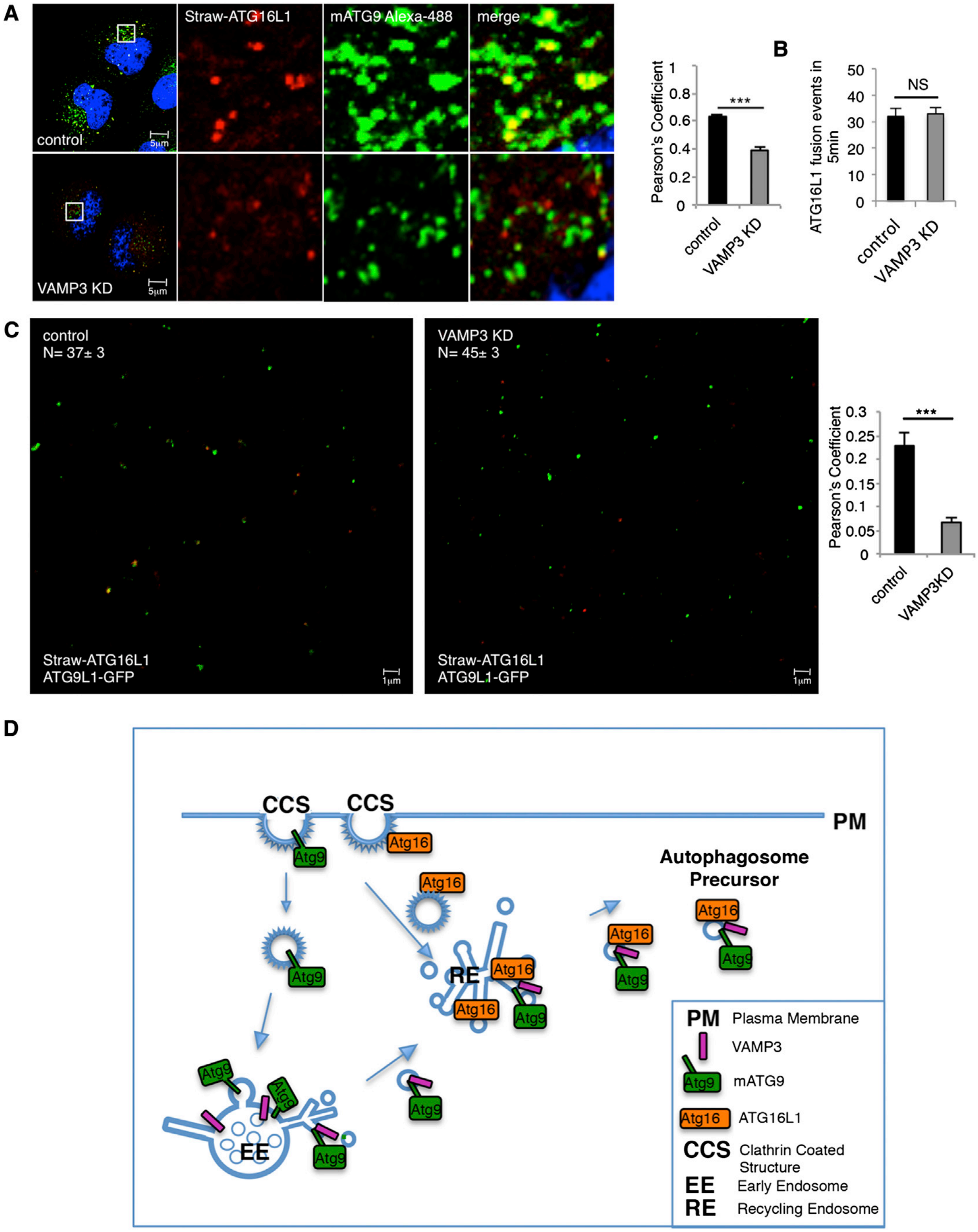

Figure 7. VAMP3 Is Required for Fusion of Vesicles Carrying mATG9 and ATG16L1

(A) HeLa cells transfected with control, VAMP3 siRNA for 4 days were transfected during the last 20 hr with mStrawberry-ATG16L1 and labeled for mATG9. The correlation between mATG9 and ATG16L1 was quantified (Pearson's coefficient). Error bar, SEM. ***$p < 0.001$.

(B) HeLa cells were transfected with control or VAMP3 siRNA and then with mStrawberry-ATG16, and 5 min movies were recorded. Homotypic fusion events between mStrawberry-ATG16 vesicles were assessed in control and VAMP3 knockdown cells. Error bar, SEM. NS, not significant.

(C) In vitro fusion assay of ATG16L1 and mATG9 vesicles. HeLa cells were transfected with control or VAMP3 siRNA for 4 days separately and, on the fourth day, either transfected with mStrawberry-ATG16L1 or ATG9L1-pEGFP. PNS of cells expressing the two constructs were mixed for 1 hr at 37C° and observed by confocal microscopy. Histogram shows the correlation of vesicles carrying ATG16L1 that fuse with vesicles carrying mATG9 (Pearson's coefficient). Error bar, SEM. ***$p < 0.001$. The total number of structures per field (ATG16L1 and ATG9L1) is not significantly different in the control and KD samples (control, 37 ± 3; VAMP3 KD, 45 ± 3; ± SEM; $p = 0.106$).

(D) Schematic diagram of mATG9 and ATG16L1 itineraries pertinent to their heterotypic fusion and autophagosome formation.

See also Figure S5 and Movies S5 and S6.

required for autophagy because VAMP3 knockdown also decreased autophagosome formation (Figure 7D). It is tempting to speculate that the VAMP3-mediated fusion of the ATG16L1 and mATG9 compartments may also be important for exit of preautophagosomal structures containing these proteins from recycling endosomes, as ATG16L1 accumulated in these structures when VAMP3 was knocked down (Figures 6D and 6E). This model suggests that ATG16L1-containing vesicles may require VAMP3/mATG9 fusion in order to be competent to exit the recycling endosomes and mature into phagophores (Figure 7D). Our data do not exclude additional sources for autophagosome membrane, and our demonstration that mATG9 and ATG16L1 meet prior to acquisition of LC3 suggests the likelihood that multiple independent membrane sources and itineraries may contribute to autophagosomes. Indeed, an important challenge for future work will be to define the distinct compartments that may contribute to autophagosome biogenesis and understand how their fusions are regulated. It is possible that such fusion events may occur in diverse cellular locations, depending on which components are examined.

EXPERIMENTAL PROCEDURES

Cell Culture

HeLa cells were cultured in Dulbecco's modified Eagle's medium (DMEM) (Sigma D6548) supplemented with 2 mM L-glutamine, 100 U/ml penicillin/streptomycin, and 10% fetal bovine serum in 5% CO_2 at 37°C.

Western Blot Analysis and Modulation of Autophagy

Cells were treated with Bafilomycin A_1 (400 nM for 4 hr or 200 nM for 16 hr) or with NH_4Cl (20 mM for 16 hr) or not treated and then lysed in Laemmli buffer. Protein samples were boiled 5–7 min at 100°C, separated by SDS-PAGE, transferred onto PVDF membranes, subjected to western blot analysis, and, finally, visualized using an ECL detection kit (GE Healthcare).

Live Imaging

HeLa cells were seeded on MatTek Petri dish (MatTek) at a density of $\sim 1.5 \times 10^5$ cells per dish. Cells were placed in HBSS (Ca^+ Mg^+) with HEPES, after which they were imaged immediately at 37°C. Five movies of 5 min were recorded for each experiment. Imaging was performed on a Zeiss Axiovert 200 M microscope with a LSM 710 confocal attachment using a 63 × 1.4 NA Plan Apochromat oil-immersion lens.

In Vitro Fusion Assay of ATG16L1 and mATG9 Vesicles

The assay was performed as described with some modifications (Moreau et al., 2011). Briefly, two postnuclear supernatants (PNS) from HeLa cells expressing either mStrawberry-ATG16L1 or ATG9L1-GFP were mixed for 60 min in the presence of an ATP regenerative system at 37°C (a control sample was left on ice). After the reaction, the samples were fixed with 2% paraformaldehyde in PBS for 15 min, centrifuged to remove the fixative (13,000 rpm, 5 min), resuspended in distilled water, and mounted with Mowiol 4-88 with DAPI on glass coverslips for confocal observation. The DAPI staining was used to identify the right focus on the coverslip.

Statistics

Significance levels for comparisons between two groups were determined with two-tailed t test. $^*p \leq 0.05$; $^{**}p \leq 0.01$; $^{***}p \leq 0.001$.

SUPPLEMENTAL INFORMATION

Supplemental Information includes Extended Experimental Procedures, five figures, and six movies and can be found with this article online at http://dx.doi.org/10.1016/j.cell.2013.08.044.

ACKNOWLEDGMENTS

We thank Dr. H.G. Wang, Dr. J.A. Mercer, Prof. DeCamilli, and Dr. B. Beaumelle for kindly providing reagents. We thank Drs. Fiona Menzies and Mariella Vicinanza for critical reading and Dr. J.D. Cooper for the statistics. We are grateful to the Wellcome Trust (Principal Research Fellowship to DCR and Strategic Grant to Cambridge Institute for Medical Research) for funding.

Received: June 14, 2012
Revised: June 28, 2013
Accepted: August 23, 2013
Published: September 12, 2013

REFERENCES

Ang, A.L., Taguchi, T., Francis, S., Fölsch, H., Murrells, L.J., Pypaert, M., Warren, G., and Mellman, I. (2004). Recycling endosomes can serve as intermediates during transport from the Golgi to the plasma membrane of MDCK cells. J. Cell Biol. *167*, 531–543.

Axe, E.L., Walker, S.A., Manifava, M., Chandra, P., Roderick, H.L., Habermann, A., Griffiths, G., and Ktistakis, N.T. (2008). Autophagosome formation from membrane compartments enriched in phosphatidylinositol 3-phosphate and dynamically connected to the endoplasmic reticulum. J. Cell Biol. *182*, 685–701.

Bonifacino, J.S., and Rojas, R. (2006). Retrograde transport from endosomes to the trans-Golgi network. Nat. Rev. Mol. Cell Biol. *7*, 568–579.

Fader, C.M., Sánchez, D.G., Mestre, M.B., and Colombo, M.I. (2009). TI-VAMP/VAMP7 and VAMP3/cellubrevin: two v-SNARE proteins involved in specific steps of the autophagy/multivesicular body pathways. Biochim. Biophys. Acta *1793*, 1901–1916.

Galli, T., Chilcote, T., Mundigl, O., Binz, T., Niemann, H., and De Camilli, P. (1994). Tetanus toxin-mediated cleavage of cellubrevin impairs exocytosis of transferrin receptor-containing vesicles in CHO cells. J. Cell Biol. *125*, 1015–1024.

Gravotta, D., Deora, A., Perret, E., Oyanadel, C., Soza, A., Schreiner, R., Gonzalez, A., and Rodriguez-Boulan, E. (2007). AP1B sorts basolateral proteins in recycling and biosynthetic routes of MDCK cells. Proc. Natl. Acad. Sci. USA *104*, 1564–1569.

Hailey, D.W., Rambold, A.S., Satpute-Krishnan, P., Mitra, K., Sougrat, R., Kim, P.K., and Lippincott-Schwartz, J. (2010). Mitochondria supply membranes for autophagosome biogenesis during starvation. Cell *141*, 656–667.

Hamasaki, M., Furuta, N., Matsuda, A., Nezu, A., Yamamoto, A., Fujita, N., Oomori, H., Noda, T., Haraguchi, T., Hiraoka, Y., et al. (2013). Autophagosomes form at ER-mitochondria contact sites. Nature *495*, 389–393.

Hayashi-Nishino, M., Fujita, N., Noda, T., Yamaguchi, A., Yoshimori, T., and Yamamoto, A. (2010). Electron tomography reveals the endoplasmic reticulum as a membrane source for autophagosome formation. Autophagy *6*, 301–303.

Jahn, R., and Scheller, R.H. (2006). SNAREs—engines for membrane fusion. Nat. Rev. Mol. Cell Biol. *7*, 631–643.

Lapierre, L.A., Kumar, R., Hales, C.M., Navarre, J., Bhartur, S.G., Burnette, J.O., Provance, D.W., Jr., Mercer, J.A., Bähler, M., and Goldenring, J.R. (2001). Myosin vb is associated with plasma membrane recycling systems. Mol. Biol. Cell *12*, 1843–1857.

Longatti, A., Lamb, C.A., Razi, M., Yoshimura, S., Barr, F.A., and Tooze, S.A. (2012). TBC1D14 regulates autophagosome formation via Rab11- and ULK1-positive recycling endosomes. J. Cell Biol. *197*, 659–675.

Luo, S., and Rubinsztein, D.C. (2010). Apoptosis blocks Beclin 1-dependent autophagosome synthesis: an effect rescued by Bcl-xL. Cell Death Differ. *17*, 268–277.

Mari, M., Griffith, J., Rieter, E., Krishnappa, L., Klionsky, D.J., and Reggiori, F. (2010). An Atg9-containing compartment that functions in the early steps of autophagosome biogenesis. J. Cell Biol. *190*, 1005–1022.

Maxfield, F.R., and McGraw, T.E. (2004). Endocytic recycling. Nat. Rev. Mol. Cell Biol. *5*, 121–132.

Mizushima, N., Kuma, A., Kobayashi, Y., Yamamoto, A., Matsubae, M., Takao, T., Natsume, T., Ohsumi, Y., and Yoshimori, T. (2003). Mouse Apg16L, a novel WD-repeat protein, targets to the autophagic isolation membrane with the Apg12-Apg5 conjugate. J. Cell Sci. *116*, 1679–1688.

Moreau, K., Ravikumar, B., Renna, M., Puri, C., and Rubinsztein, D.C. (2011). Autophagosome precursor maturation requires homotypic fusion. Cell *146*, 303–317.

Moreau, K., Ravikumar, B., Puri, C., and Rubinsztein, D.C. (2012). Arf6 promotes autophagosome formation via effects on phosphatidylinositol 4,5-bisphosphate and phospholipase D. J. Cell Biol. *196*, 483–496.

Ohsumi, Y., and Mizushima, N. (2004). Two ubiquitin-like conjugation systems essential for autophagy. Semin. Cell Dev. Biol. *15*, 231–236.

Orsi, A., Razi, M., Dooley, H.C., Robinson, D., Weston, A.E., Collinson, L.M., and Tooze, S.A. (2012). Dynamic and transient interactions of Atg9 with autophagosomes, but not membrane integration, are required for autophagy. Mol. Biol. Cell *23*, 1860–1873.

Prekeris, R., Klumperman, J., Chen, Y.A., and Scheller, R.H. (1998). Syntaxin 13 mediates cycling of plasma membrane proteins via tubulovesicular recycling endosomes. J. Cell Biol. *143*, 957–971.

Puri, C., Chibalina, M.V., Arden, S.D., Kruppa, A.J., Kendrick-Jones, J., and Buss, F. (2010). Overexpression of myosin VI in prostate cancer cells enhances PSA and VEGF secretion, but has no effect on endocytosis. Oncogene *29*, 188–200.

Ravikumar, B., Vacher, C., Berger, Z., Davies, J.E., Luo, S., Oroz, L.G., Scaravilli, F., Easton, D.F., Duden, R., O'Kane, C.J., and Rubinsztein, D.C. (2004). Inhibition of mTOR induces autophagy and reduces toxicity of polyglutamine expansions in fly and mouse models of Huntington disease. Nat. Genet. *36*, 585–595.

Ravikumar, B., Moreau, K., Jahreiss, L., Puri, C., and Rubinsztein, D.C. (2010). Plasma membrane contributes to the formation of pre-autophagosomal structures. Nat. Cell Biol. *12*, 747–757.

Ren, M., Xu, G., Zeng, J., De Lemos-Chiarandini, C., Adesnik, M., and Sabatini, D.D. (1998). Hydrolysis of GTP on rab11 is required for the direct delivery of transferrin from the pericentriolar recycling compartment to the cell surface but not from sorting endosomes. Proc. Natl. Acad. Sci. USA *95*, 6187–6192.

Renna, M., Schaffner, C., Winslow, A.R., Menzies, F.M., Peden, A.A., Floto, R.A., and Rubinsztein, D.C. (2011). Autophagic substrate clearance requires activity of the syntaxin-5 SNARE complex. J. Cell Sci. *124*, 469–482.

Rubinsztein, D.C., Cuervo, A.M., Ravikumar, B., Sarkar, S., Korolchuk, V., Kaushik, S., and Klionsky, D.J. (2009). In search of an "autophagomometer". Autophagy *5*, 585–589.

Rubinsztein, D.C., Mariño, G., and Kroemer, G. (2011). Autophagy and aging. Cell *146*, 682–695.

Sönnichsen, B., De Renzis, S., Nielsen, E., Rietdorf, J., and Zerial, M. (2000). Distinct membrane domains on endosomes in the recycling pathway visualized by multicolor imaging of Rab4, Rab5, and Rab11. J. Cell Biol. *149*, 901–914.

Stenmark, H., Parton, R.G., Steele-Mortimer, O., Lütcke, A., Gruenberg, J., and Zerial, M. (1994). Inhibition of rab5 GTPase activity stimulates membrane fusion in endocytosis. EMBO J. *13*, 1287–1296.

Stoorvogel, W., Oorschot, V., and Geuze, H.J. (1996). A novel class of clathrin-coated vesicles budding from endosomes. J. Cell Biol. *132*, 21–33.

Thompson, H.M., and McNiven, M.A. (2006). Discovery of a new 'dynasore'. Nat. Chem. Biol. *2*, 355–356.

Tosoni, D., Puri, C., Confalonieri, S., Salcini, A.E., De Camilli, P., Tacchetti, C., and Di Fiore, P.P. (2005). TTP specifically regulates the internalization of the transferrin receptor. Cell *123*, 875–888.

Ullrich, O., Reinsch, S., Urbé, S., Zerial, M., and Parton, R.G. (1996). Rab11 regulates recycling through the pericentriolar recycling endosome. J. Cell Biol. *135*, 913–924.

van der Vaart, A., Griffith, J., and Reggiori, F. (2010). Exit from the Golgi is required for the expansion of the autophagosomal phagophore in yeast Saccharomyces cerevisiae. Mol. Biol. Cell *21*, 2270–2284.

Warren, R.A., Green, F.A., and Enns, C.A. (1997). Saturation of the endocytic pathway for the transferrin receptor does not affect the endocytosis of the epidermal growth factor receptor. J. Biol. Chem. *272*, 2116–2121.

Warren, R.A., Green, F.A., Stenberg, P.E., and Enns, C.A. (1998). Distinct saturable pathways for the endocytosis of different tyrosine motifs. J. Biol. Chem. *273*, 17056–17063.

Wilcke, M., Johannes, L., Galli, T., Mayau, V., Goud, B., and Salamero, J. (2000). Rab11 regulates the compartmentalization of early endosomes required for efficient transport from early endosomes to the trans-golgi network. J. Cell Biol. *151*, 1207–1220.

Wiley, H.S. (1988). Anomalous binding of epidermal growth factor to A431 cells is due to the effect of high receptor densities and a saturable endocytic system. J. Cell Biol. *107*, 801–810.

Yamazaki, Y., Schönherr, C., Varshney, G.K., Dogru, M., Hallberg, B., and Palmer, R.H. (2013). Goliath family E3 ligases regulate the recycling endosome pathway via VAMP3 ubiquitylation. EMBO J. *32*, 524–537.

Ylä-Anttila, P., Vihinen, H., Jokitalo, E., and Eskelinen, E.L. (2009). 3D tomography reveals connections between the phagophore and endoplasmic reticulum. Autophagy *5*, 1180–1185.

Young, A.R., Chan, E.Y., Hu, X.W., Köchl, R., Crawshaw, S.G., High, S., Hailey, D.W., Lippincott-Schwartz, J., and Tooze, S.A. (2006). Starvation and ULK1-dependent cycling of mammalian Atg9 between the TGN and endosomes. J. Cell Sci. *119*, 3888–3900.

EXTENDED EXPERIMENTAL PROCEDURES

Antibodies and Reagents

The antibodies used include: rabbit anti-RAB11 (Invitrogen-715300), rabbit anti-ATG16L1 (CosmoBio Co. LTP- TMD-PH-ATG16L; Cell Signaling: 8089S), ATG12 (Cell Signaling: 2010S), mouse anti-tubulin (SIGMA), rabbit anti-LC3 for Western blotting (Novus biological- NB100-2220), mouse anti-HA (Covance MMS-10l), rabbit anti-ATG9 (ABCAM-Ab108338 and SIGMA-A0732), sheep anti-TGN46 (AbD Serotec AH-P500), mouse anti-human Transferrin Receptor (ATCC-5E9C11), human transferrin Alexa-555, Alexa-647 and Alexa-488 (Invitrogen), mouse anti-EEA1 (ABCAM-ab70521), rabbit anti-Clathrin (ABCAM- ab21679), Cholera toxin HRP (Invitrogen), mouse anti-AP2 μ2 (BD AP-50) and anti-HRP (Sigma-P7899), rabbit anti-VAMP3 was a kind gift from A.A. Peden. All the Alexa-conjugated secondary antibodies are from INVITROGEN. Protein-A gold is from CMC (Utrecht- NL).

Bafilomycin A_1 and Dynasore are from SIGMA.

Plasmids

pmStrawberry-ATG16L1, pEGFP-ATG16L1 and httQ74-HA have been described elsewhere (Cadwell et al., 2008; Ravikumar et al., 2002). ATG9L1-pEGFP was a kind gift from Dr Y. Takahashi, VAMP3-HA was a kind gift from Dr. A.A. Peden and pEGFP-LC3 was a kind gift from Tamotsu Yoshimori, pEGFP-MVb tail was a kind gift from Dr. J.A. Mercer and Dynamin K44A pCDNA was a kind gift from Dr. B. Beaumelle.

RAB11 was cloned in pEGFP and mCherry C1 into the Xho BamH1 sites. The constructs were validated by sequencing.

Cell Transfection

The cells were seeded at 1-2 $x10^5$ per 6-well and transfections were performed using LipofectAMINE 2000 for siRNA and Myrus Bio *Trans*IT -2020, according to the manufacturer's instructions, using 80 nM siRNA (AP2 μ2 siRNA custom oligos that we used previously in Ravikumar et al. [2010]) and were compared with control siRNA. ATG16L1-pEGFP and ATG16L1-mStrawberry were transfected in a concentration of 0.3μg per well, httQ74-HA at 0.5 μg and the other constructs at 1μg.

VAMP3 was silenced using oligo A (GGCAGGCGCUUCUCAAUUU) or alternatively oligo B (GGAUUACUGUUUCUGGUUAU) and C (GAGUUAACGUGGACAAGGU). All the SMARTpool oligos for the screening were from Dharmacon.

Aggregate Quantification

The httQ74-HA aggregation was detected by immunofluorescence (primary HA antibody). The proportion of transfected cells with aggregates was scored (approx. 500 cells per coverslip). Experiments were performed blinded and in triplicate in at least three independent experiments. Statistics for aggregation assays were calculated as odds ratios (the ratio of cells containing aggregates in each condition).

Immunofluorescence Microscopy

HeLa cells were grown on coverslips at confluency of 25% were fixed in 4% paraformaldehyde for 5 min and then permeabilized with 0.1% Triton. 1% BSA in PBS was used for blocking and primary and secondary antibodies. A Zeiss LSM710 confocal microscope was used for fluorescent confocal analysis. All confocal images were taken with a 63 × oil-immersion lens. ImageJ (size and number of vesicles analysis) and Volocity software (PerkinElmer) (colocalization analysis using Pearson's Coefficient or Manders' Coefficient) were used for further analysis and processing of confocal images. The Pearson's Coefficient was used to measure the correlation between the signals from two different markers/proteins. The Manders' Coefficient was used to quantify the localization of protein A in compartment B under different conditions. A minimum of 20 cells were examined each condition. All experiments have been repeated at least three times. The background was fixed for all within-experiment analyses (for Mander's).

TIRF and Dynasore Treatment

HeLa cells were treated with Dynasore 100 μM for 2 hr in serum-free medium, or transfected for 16h with Dynamin K44A mutant or AP2 siRNA KD (5th day of treatment) fixed and labeled with anti-ATG9 antibody.

The samples were analyzed by total internal reflection fluorescence (TIRF) microscopy (TIRF 3, Carl Zeiss MicroImaging Inc.).

Immunogold Electron Microscopy

HeLa cells were transfected with GFP-ATG16L1 for 24h. The cells were starved 1h in HBSS, loaded with anti-TfR antibody (5E9D11) for 1h and then fixed with a mixture of 2% paraformaldehyde and 1% acrolein in phosphate buffer (pH. 7.4) for 2h, at room temperature. Cells were then prepared for ultrathin cryosectioning and immunogold-labeled, as previously described (Puri, 2009; Slot and Geuze, 2007). Briefly, fixed cells were washed once in PBS/0.02 M glycine, after which cells were scraped in 12% gelatin in PBS and embedded in the same solution. The cell-gelatin was cut into 1 mm blocks, infiltrated with 15% PVP in 1.7 M sucrose at 4°C overnight, mounted on aluminum pins and frozen in liquid nitrogen. Ultrathin cryosections were picked up in a mixture of 50% sucrose and 50% methylcellulose and incubated with primary antibodies (ATG16L1, ATG9 and rabbit anti-mouse to recognize TfR) followed by protein A gold (Utrecht). Double and triple labeling was performed as previously described (Puri et al., 2005; Slot and Geuze, 2007).

We optimized double and triple labeling as follows. Briefly, we first performed the three separate single labelings to assess the specificity and the localization of the three different proteins (Clathrin, mATG9 and ATG16L1). Then we performed three sets of double labeling (mATG9/Cla, ATG16L1/Cla, mATG9/ATG16L1) and the opposite sequential combinations (Cla/mATG9, Cla/ATG16L1, ATG16L1/mATG9). This helps us determine the correct sequential order for the antibody labeling. The protein with more abundant labeling is used first (with gold 5nm), which in our case is clathrin. We discovered from the double immunolabeling that mATG9 is the weakest antibody and thus we labeled this last (as the antibody and the gold tend to detach during the multiple immunolabeling procedures). Thus, we decided on the order Clathrin 5nm/ATG16 10 nm/ ATG9 15 nm. The protein A gold is always used from the smallest size to the largest. After each labeling step, the reaction was fixed 5 min with 1% glutaraldehyde in PBS to quench the free antibodies and stabilize the reaction. Of course, with these multiple steps one acquires data with single as well as double labeling and one can monitor for any possible labeling abnormalities that may occur with the sequential process. As an example, we now show single, double and triple labeling in Figures 1F, 1H, and 1I, respectively.

Morphometric analysis was performed directly on the pictures by counting the tubulo-vesicular structures carrying both markers (ATG16L1 and RAB11), compared with the total ATG16L1-labeled structures (Figure 2C).

FACS-Based Endocytosis Assays

Transferrin-recycling assay was performed as previously described (Peden et al., 2004). Briefly, the cells were incubated for 30 min at 37°C in the continuous presence of Tf–Alexa-Fluor-647. Cells were then washed and incubated at 37°C in media supplemented with 100 μg/ml unlabeled transferrin for various times before fixation in 4% paraformaldehyde in PBS. Cell-associated Tf–Alexa-Fluor-647 was determined by FACS analysis using BD FACSCalibur flow cytometer (BD Biosciences) and FlowJo software (Tree Star Inc.).

Biotinylation of Cell Surface Proteins

HeLa cells grown on 6-well plates were rinsed twice with ice-cold PBS^{2+} (containing 1 mM $MgCl_2$ and 0.1 mM $CaCl_2$). Cells were subsequently incubated with freshly prepared ice-cold NHS-LC-Biotin (Pierce) solution (1 mg/ml in ice-cold PBS, 1 mM $MgCl_2$, 2 mM $CaCl_2$, 150 mM NaCl) for 60 - 90 min at 4°C with gentle agitation. Unreacted NHS-LC-Biotin was then quenched by washing the cells twice with ice-cold quenching buffer (PBS, 1 mM $MgCl_2$, 0.1 mM $CaCl_2$, 100 mM glycine) for 20 min at 4°C. After quenching, cells were rinsed twice with ice-cold PBS++ and were scraped in RIPA buffer [50 mM Tris-HCl, pH 7.4, 150 mM NaCl, 5 mM EDTA, 1% NP-40, 0.5% Sodium Deoxycholate, 0.1% SDS and supplemented with phosphatase and protease inhibitor cocktails]. After 30 min on ice and occasional brief vortexing, lysates were cleared by centrifugation at 16 000 g for 10 min at 4°C. Samples were then transferred to new tubes containing 40 μl of streptavidin-agarose beads (Pierce). After 2 hr of incubation at 4°C under agitation, the beads were washed three times with RIPA buffer and one time with PBS. The final pellets were resuspended in 40 μl of 2 × Laemmli buffer and denatured at 100°C for 5 min. The beads were pelleted and the solubilized proteins were separated by SDS-PAGE, which was silver stained or transferred onto PVDF membranes and probed with antibodies against ATG9 (ABCAM). Nonspecific binding of unlabeled proteins was assessed by including a control condition in which the biotinylation buffer did not contain biotin.

SUPPLEMENTAL REFERENCES

Cadwell, K., Liu, J.Y., Brown, S.L., Miyoshi, H., Loh, J., Lennerz, J.K., Kishi, C., Kc, W., Carrero, J.A., Hunt, S., et al. (2008). A key role for autophagy and the autophagy gene Atg16l1 in mouse and human intestinal Paneth cells. Nature *456*, 259–263.

Peden, A.A., Schonteich, E., Chun, J., Junutula, J.R., Scheller, R.H., and Prekeris, R. (2004). The RCP-Rab11 complex regulates endocytic protein sorting. Mol. Biol. Cell *15*, 3530–3541.

Puri, C. (2009). Loss of myosin VI no insert isoform (NoI) induces a defect in clathrin-mediated endocytosis and leads to caveolar endocytosis of transferrin receptor. J. Biol. Chem. *284*, 34998–35014.

Puri, C., Tosoni, D., Comai, R., Rabellino, A., Segat, D., Caneva, F., Luzzi, P., Di Fiore, P.P., and Tacchetti, C. (2005). Relationships between EGFR signaling-competent and endocytosis-competent membrane microdomains. Mol. Biol. Cell *16*, 2704–2718.

Ravikumar, B., Duden, R., and Rubinsztein, D.C. (2002). Aggregate-prone proteins with polyglutamine and polyalanine expansions are degraded by autophagy. Hum. Mol. Genet. *11*, 1107–1117.

Slot, J.W., and Geuze, H.J. (2007). Cryosectioning and immunolabeling. Nat. Protoc. *2*, 2480–2491.

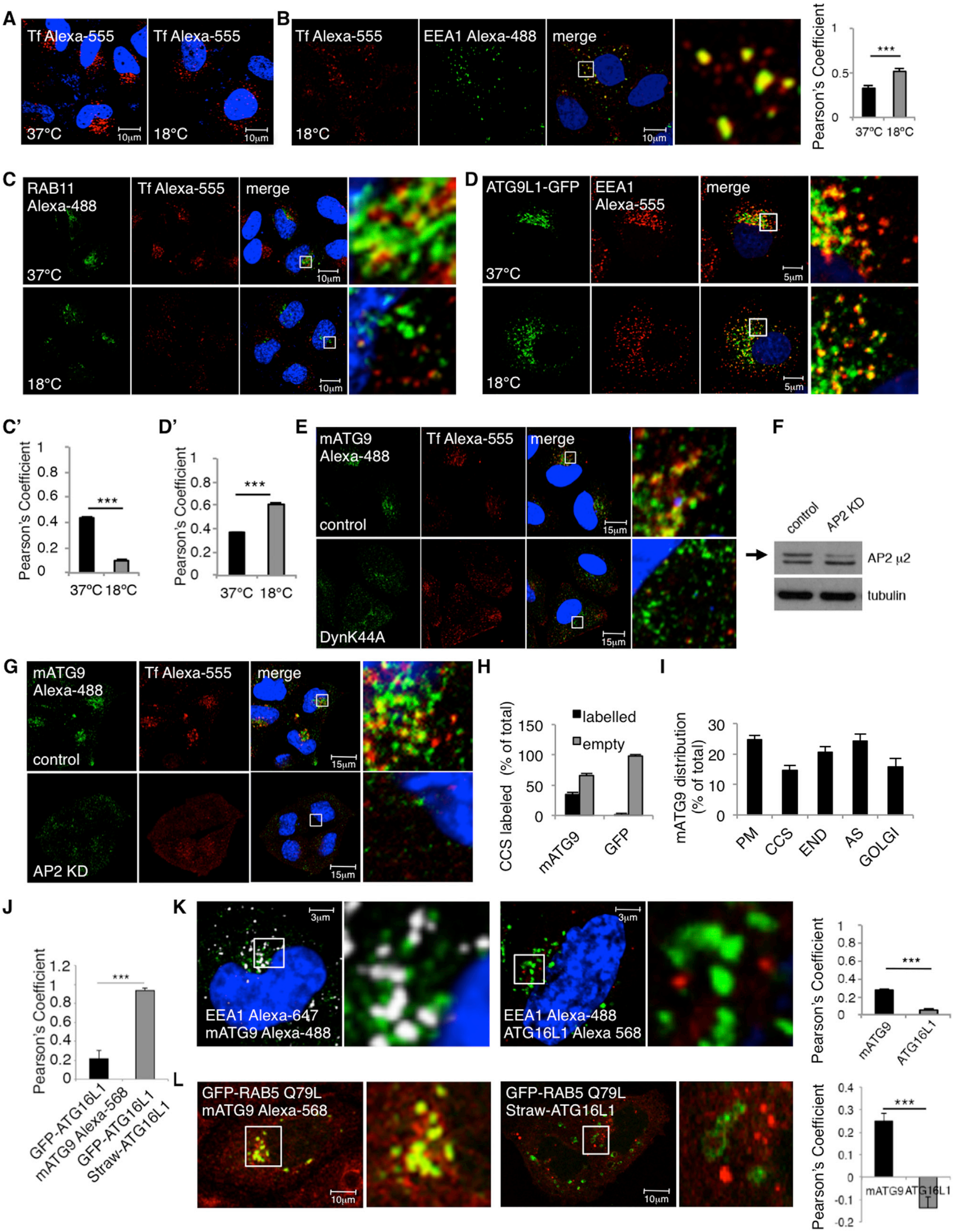

(legend on next page)

Figure S1. mATG9 Is Trafficked from the Plasma Membrane to Early Endosomes, Related to Figure 1

(A) HeLa cells were incubated 1 hr at 18°C or 37°C and loaded for 1h with transferrin Alexa-555. The pictures represent representative fields of transferrin localization at 18°C or 37°C. Transferrin localization appears more disperse at 18°C.

(B) HeLa cells treated with the same protocol described in (A) were fixed and labeled with anti-EEA1 antibody (early endosome marker). At 18°C transferrin accumulates in early endosomes. The Pearson's Coefficient between transferrin and EEA1 was quantified in the histogram. Error Bar = SEM. *** = $p < 0.001$.

(C and C′) HeLa cells treated as in (A) were labeled with anti-RAB11 antibody – RAB11 is the most commonly used marker for recycling endosomes. At 18°C transferrin is no longer able to reach the recycling endosome and therefore shows less colocalization with RAB11. The Pearson's Coefficient between transferrin and RAB11 was quantified in the histogram. Error Bar = SEM. *** = $p < 0.001$.

(D and D′) HeLa cells treated as in (A) were transfected with ATG9L1-GFP and labeled with anti-EEA1- EEA1 is the most commonly used marker for early endosomes. At 18°C mATG9 is no longer able to exit from the endosome and therefore shows more colocalization with EEA1. The Pearson's Coefficient between mATG9 and EEA1 was measured in the histogram. Error Bar = SEM. *** = $p < 0.001$.

(E) HeLa cells were transfected with dynamin dominant-negative mutant (K44A) for 20 hr, loaded for 30 min with transferrin Alexa-555 and labeled for mATG9. The pictures show a re-localization of mATG9 from perinuclear locations to a more peripheral area of the cell (close to plasma membrane).

(F and G) HeLa cells were RNA silenced for AP2 (μ2) for 5 days to inhibit clathrin-mediated endocytosis and treated as in (E). A control blot shows the level of AP2 (μ2) reduction (the arrow shows the AP2 (μ2) band).

(H) HeLa cells transfected or not with empty pEGFP vector were processed for immunogold labeling on cryosections and labeled with anti-ATG9 antibody. The GFP protein is cytoplasmic and is not associated with clathrin-coated structures. This marker was used as negative control for the specificity of mATG9 localization on clathrin-coated structures. Error bar = SEM.

(I) HeLa cells were fixed in basal conditions and processed for immunogold labeling on cryosections and stained with anti-mATG9 antibody. 10 cell profiles in two different experiments were considered by counting the number of gold particles (mATG9) in different compartments (PM: plasma membrane; CCS: clathrin-coated structures; END: endosomes; AS: autophagic structures; GOLGI: Golgi). The percentage of mATG9 localization in different compartments was quantified in the histogram and expressed as percentage of the total. Error bar = SEM.

(J) The histogram shows the quantification expressed as Pearson's Coefficient of the experiment shown in Figure 1 G. Error bar = SEM. *** = $p < 0.001$.

(K) HeLa cells were fixed and labeled with EEA1 (early endosomes) and ATG16L1or mATG9. The Pearson's Coefficient between EEA1 and ATG16L1 or mATG9 was quantified in the histogram. Error bar = SEM. *** = $p < 0.001$.

(L) HeLa cells were transfected with GFP-RAB5 constitutively-active mutant (Q79L) for 20 hr and mStraw-ATG16L1, or transfected with GFP-RAB5 Q79L and labeled with anti-mATG9 antibody. This constitutively-active mutant induces aberrant fusion of early endosomes. The Pearson's Coefficient between RAB5 Q79L and mATG9 or ATG16L1 was quantified in the histogram. Error bar = SEM. *** = $p < 0.001$.

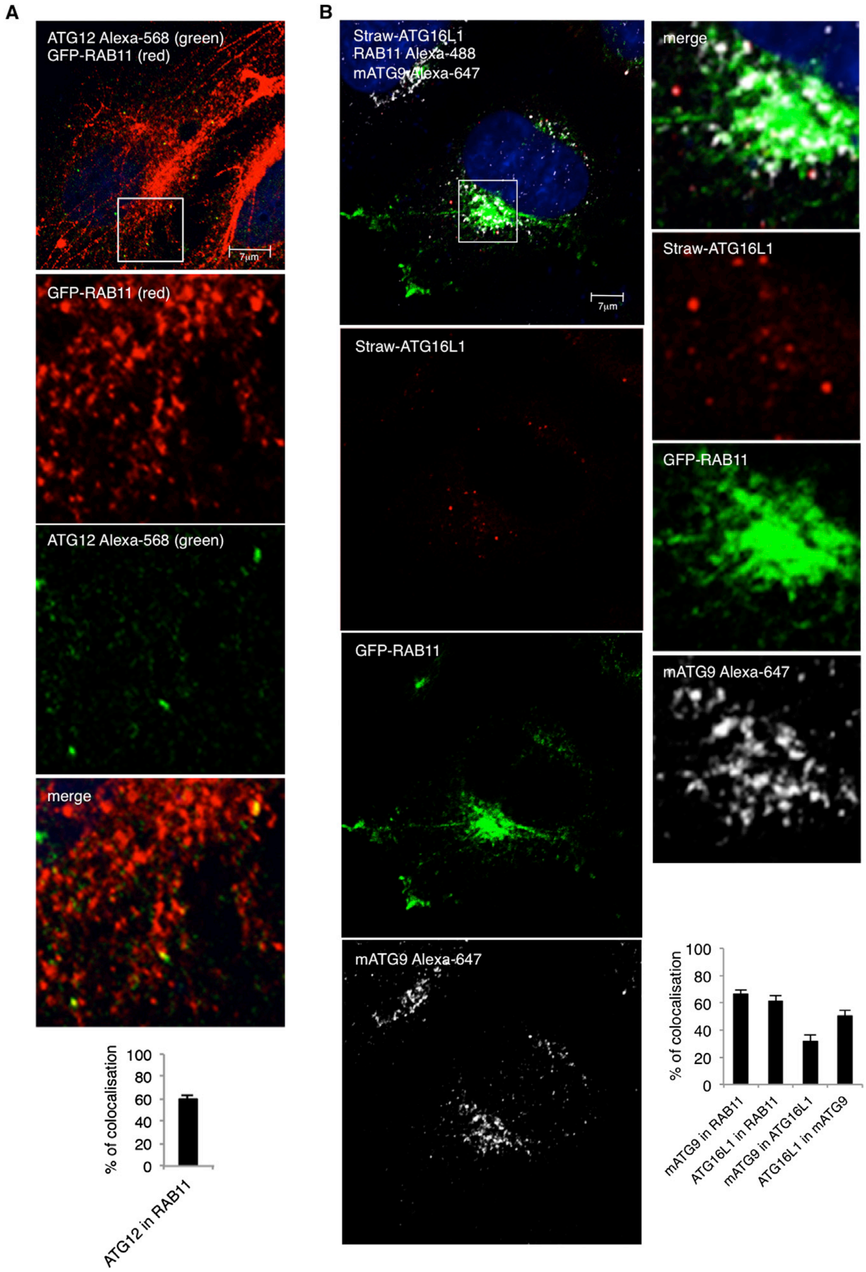

Figure S2. ATG12 and ATG16L1 Localize in Recycling Endosomes, Related to Figure 2

(A) HeLa cells where transfected with GFP-RAB11 and labeled for ATG12 (the pictures show pseudo-colors). The amount of colocalization of ATG12 with RAB11 (recycling endosomes) is shown in the histogram expressed as Manders' Coefficient. Error bar = SEM.

(B) HeLa cells were transfected with pmStrawberry-ATG16L1 and pEGFP-RAB11. 20 hr later, the cells were fixed in basal conditions and stained for mATG9. The histogram shows the relative colocalization between RAB11-ATG16L1, RAB11-mATG9 and ATG16L1-mATG9 expressed as Manders' Coefficient. Error bars = SEM.

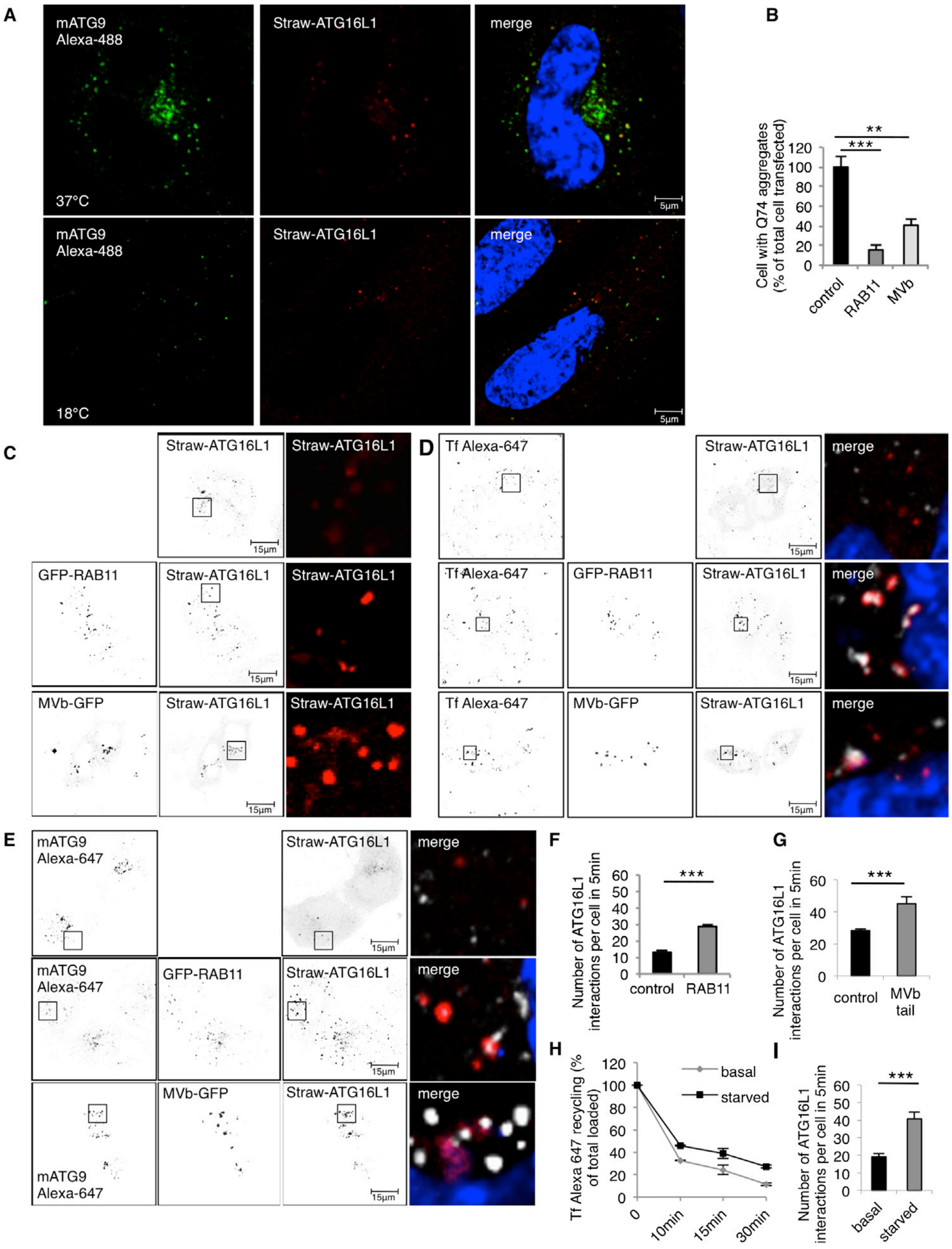

Figure S3. Recycling Endosome Trafficking Regulates ATG16L1/mATG9 Vesicle Fusion, Related to Figure 3

(A) This figure shows the individual channels used for the composites in Figure 3H.

(B) HeLa cells were transfected with HA-Q74 huntingtin and RAB11-EGFP or MVb tail-EGFP or EGFP empty vector constructs for 48 hr. The Q74 huntingtin aggregates were identified with anti-HA antibody. Error bar = SD from 3 different experiments. *** = $p < 0.001$; ** = $p < 0.01$.

(C–E) This figure shows the individual channels (in negative to enable clear visualization of the vesicles) used for the composites in Figures 3J–3L.

(F–G) HeLa cells were transfected with mCherry-RAB11 and pEGFP-ATG16L1 (F) or mStrawberry-ATG16L1 and EGFP-MVb tail (G). 5 movies of 5 min each movies were recorded each experiment. The histograms in F and G show the interactions (short and long-lived homotypic fusions) between ATG16L1 vesicles in control or RAB11- or MVb-overexpressing conditions. Error Bar = SEM. *** = $p < 0.001$.

(H) HeLa cells were amino acid- and serum-starved for 4 hr (or maintained in full medium) and loaded for 30 min with transferrin Alexa-647. Unlabeled transferrin was then used to induce recycling for different times. The samples were then fixed wit 4% Paraformaldehyde and analyzed by FACS. The values in the graph represent the amounts of intracellular Tf—higher values correspond to less recycling. Error Bar = SD. Post hoc Test Anova < 0.05.

(I) HeLa cells were transfected with pEGFP-ATG16L1. 5 movies of 5 min each were recorded in cells incubated with HBSS (starvation) or full medium. The histogram shows the homotypic interactions between ATG16L1 vesicles in control or starved cells. A minimum of 20 cells were examined each condition. Error Bar = SEM. *** = $p < 0.001$. See also Movies S3 and S4.

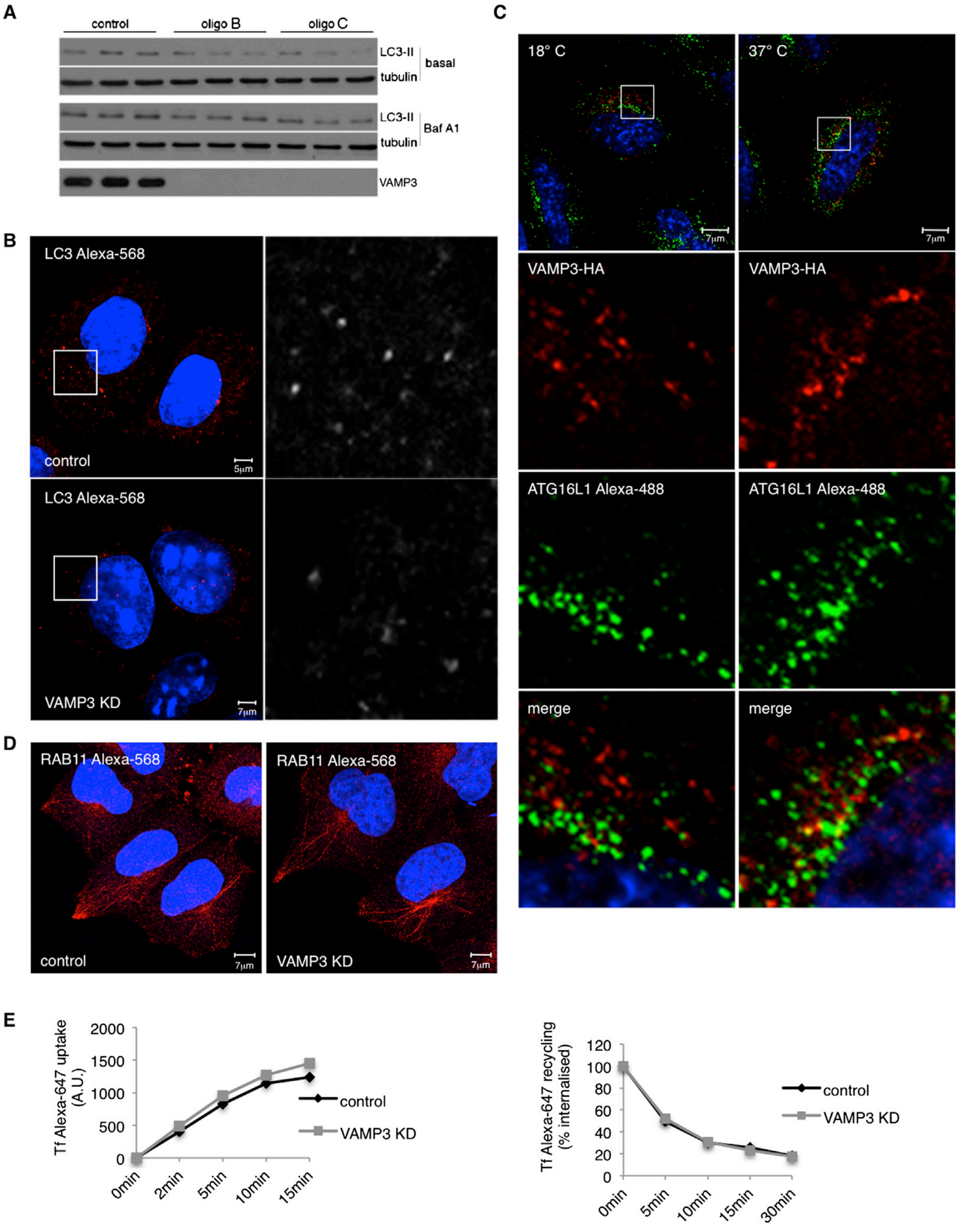

Figure S4. VAMP3 Depletion Affects Autophagy and Does Not Affect General Endocytosis, Related to Figure 5

(A) HeLa cells were transfected with oligos B (GGAUUACUGUUUCUGGUUAU) and C (GAGUUAACGUGGACAAGGU) for VAMP3, or with control oligo and blotted for LC3-II in presence (Baf A_1) or absence of Bafilimycin A_1 (basal). Tubulin was used as loading control.

(B) HeLa cells transfected with control or VAMP3 siRNA (oligo A) for 4 days were subjected to immunocytochemistry for endogenous LC3.

(C) HeLa cells were transfected for 20h with VAMP3-HA incubated 1 hr at 18°C or 37°C and labeled for endogenous ATG16L1. The colocalization at 18°C or 37°C is shown in Figure 5F.

(D) HeLa cells transfected with control or VAMP3 siRNA were labeled for endogenous RAB11. E) HeLa cells treated with control or VAMP3 siRNA were loaded with transferrin Alexa-647 for different times (0, 2, 5, 10 and 15 min) or loaded for 30 min with transferrin Alexa-647, washed and incubated for different times (5, 10, 15 and 30 min) with unlabeled transferrin for the recycling assay. Error bar = SD. Loss of VAMP3 does not affect both transferrin uptake and recycling.

Cell

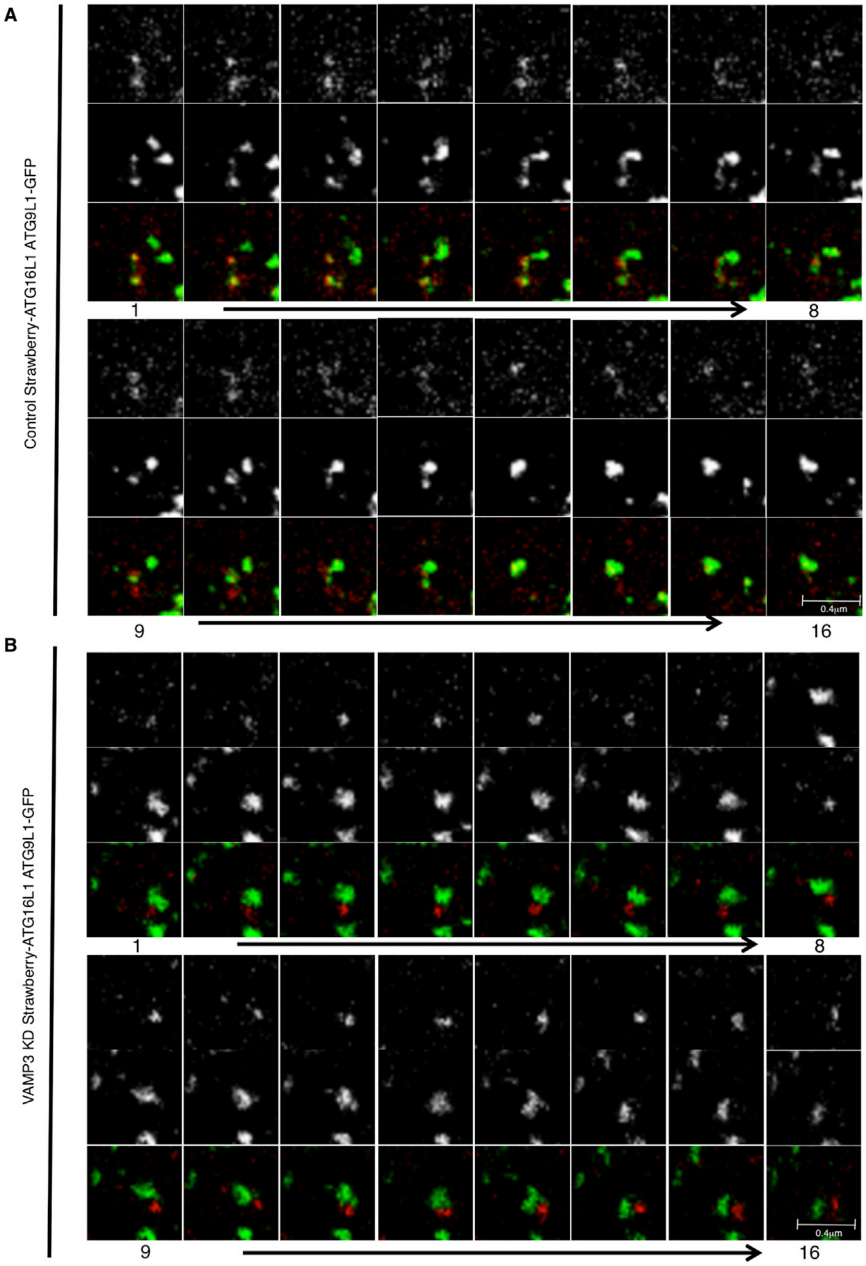

Figure S5. VAMP3 Depletion Affects ATG16L1/mATG9 Vesicle Fusion, Related to Figure 7

(A and B) HeLa cells transfected with control (A), VAMP3 siRNA (B) for 4 days were transfected for 20 hr with mStrawberry-ATG16L1 and ATG9L1-pEGFP. Representative images from 5 min Movies are shown. (See also Movies S5 and S6.)
